# System-wide investments generate health and enhance HIV, TB and malaria control in Malawi

**DOI:** 10.1038/s41467-026-77306-5

**Published:** 2026-09-04

**Authors:** Tara D. Mangal, Sakshi Mohan, Margherita Molaro, Joseph Collins, Tim Colbourn, Eva Janoušková, Rachel E. Murray-Watson, Dominic Nkhoma, Andrew Phillips, Bingling She, Pakwanja D. Twea, Simon Walker, Paul Revill, Timothy B. Hallett

**Affiliations:** 1 MRC Centre for Global Infectious Disease Analysis, School of Public Health, Imperial College London, London, UK; 2 Centre for Health Economics, University of York, York, UK; 3 Institute for Global Health, University College London, London, UK; 4 Health Economics and Policy Unit, Kamuzu University of Health Sciences, Blantyre, Malawi; 5 Department of Planning and Policy Development, Ministry of Health and Population, Lilongwe, Malawi

**Keywords:** Epidemiology, Health care economics, Infectious diseases

## Abstract

Global health initiatives have expanded access to HIV, tuberculosis, and malaria services in low-income countries, yet these programmes often operate within fragile health systems where shortages of healthcare workers and essential medicines limit their effectiveness and sustainability. Evaluating investments in health system capacity remains challenging because their benefits are difficult to attribute. Here, using a dynamic microsimulation model of Malawi’s health system, we show that health system strengthening investments alone could avert 11.4% of disability-adjusted life years between 2025 and 2035, delivering a return of $26.1 per dollar invested. Combining system strengthening with HIV, tuberculosis, and malaria programme expansion produces three times greater health impact than disease-focused investment alone, delivering a return of $11.7 per dollar invested compared with $5.4 for disease-focused expansion alone. These findings support a shift toward integrated global health financing strategies that combine health system strengthening with disease-targeted programmes.

## Abstract (web summary)

A modelling study of Malawi’s health system shows that combining health system strengthening with disease-targeted programmes delivers twice the return on investment and three times the health impact of disease-focused investment alone.

## Introduction

Over the past thirty years, global health initiatives (GHIs) have directed substantial resources into programs targeting HIV/AIDS, tuberculosis, and malaria (HTM)^1^. These efforts, spearheaded by organizations such as the Global Fund to Fight AIDS, Tuberculosis and Malaria, Gavi, the Vaccine Alliance, and the President’s Emergency Plan for AIDS Relief, have significantly expanded access to essential medicines, diagnostics, and health services, resulting in large improvements in population health. By 2023, new HIV infections had dropped to their lowest levels since the 1980s, and nearly 31 million people were receiving lifesaving antiretroviral therapy (ART)^2^. TB treatment coverage in Global Fund-supported countries reached 70%, up from 45% in 2010^3^. Malaria control efforts prevented 2.1 billion cases and 11.7 million deaths globally between 2000 and 2022^4^. Together, these initiatives have contributed to a rise in life expectancy in sub-Saharan Africa, from 56.3 years in 2010 to 61.1 years in 2023^5^.

These disease-specific investments often led to fragmented service delivery, straining fragile health systems^6,7^. Recognizing this, GHIs began incorporating health system strengthening (HSS) into their funding strategies, aiming to enhance service delivery, supply chains, and health information systems. However, the majority of HSS funding remains tethered to disease-specific interventions rather than broader, system-wide investments^1,8^, often leading to inefficient parallel health system structures^9,10^. Historical preferences for HTM-focussed programs have also reflected funding mechanics—notably short donor cycles with an emphasis on near-term, measurable impacts and the use of off-budget or parallel delivery channels—which shape what is financed and how it is implemented.

A major contributor to this narrow investment focus is a critical gap in empirical evidence on the health impact and efficiency of health system investments, which present an alternative to the better-evidenced disease-focussed investments. Arguments for system-strengthening are longstanding and supported by multiple evidence streams^7,11,12^. Early analyses also highlighted that investments targeted at priority diseases could be designed to strengthen underlying delivery functions rather than operate solely within program silos^13^. These investments describe how delivery is structured, whether resources and accountability are focussed in HTM programs, in cross-cutting enablers, or in a combination, rather than denoting spend levels alone. The challenge in measuring the health impact of system-wide investments arises because their benefits are mediated through improvements in many different aspects of healthcare delivery rather than direct, easily measurable and attributable health outcomes^14^. This evidence gap and its effect on funding preferences threatens the efficiency and effectiveness of health sector investments by both GHIs and country governments, particularly in settings with severely constrained health systems^10^.

Recent studies highlight that health system constraints, particularly human resources for health (HRH) shortages and weak supply chains, can impede the scalability of HTM programs^15–17^. Evidence increasingly points to the synergistic value of investments in core health system functions and HTM programs^11,12^. Such approaches, which integrate HRH strengthening, improved availability of consumables, and disease-specific interventions, have been shown to enhance both efficiency and equity, yielding substantially greater health benefits than isolated approaches^18–20^. Despite this, system-wide and combined disease-focused and system investment approaches are underexplored, particularly in high-burden LMIC settings where health system challenges are most acute.

Malawi exemplifies the challenges faced by countries with constrained health systems. Severe shortages of HRH^21–23^, along with fragile supply chains and frequent stock-outs of essential consumables^24,25^, undermine service delivery and limit the scalability of health interventions and impact^17^. Amid tightening fiscal space, modelling has been used to characterise the health consequences of declining aid and to identify protection and efficiency options^23^. Compounding these challenges, Malawi bears a high burden of both HTM and non-HTM diseases, including acute lower respiratory infections, diarrhoeal diseases and non-communicable diseases, which disproportionately affect marginalized populations^26^.

Here, we develop a dynamic modelling framework to assess the impact of alternative investment strategies over 11 years (2025–2035) on healthcare service delivery and disease burden in Malawi, explicitly accounting for real-world constraints such as workforce shortages and supply chain disruptions. Utilizing the Thanzi La Onse (TLO) model^25^, an individual-based simulation of population health and healthcare interactions, we evaluate three investment approaches: HTM-focussed (HTM), health system strengthening through supply chains and human resources for health (HSS), and combined (HTM alongside HSS) and compare these to a *Baseline* scenario where health system capacity and the coverage of HIV, TB and malaria disease programs remains at 2024 levels. The HTM-focussed approach encompassed service coverage, diagnostics, and treatment adherence (Table 1). The HSS approach included different levels (1%, 4.85%, 6%) of annual scale-up of all HRH, improvements in the availability of consumables to benchmark levels, and a comprehensive *HSS Expansion Package* that combines these components (Table 1 and Supplementary Fig. 2). This provides a quantitative assessment of the health impact and value-for-money of HSS investments, their comparison with HTM-focussed investments, and the potential synergies when these approaches are combined. Health outcomes are measured using disability-adjusted life years (DALYs), mortality, and health service coverage.

**Table 1:** Description of scenarios

**Health system strengthening (HSS) investment approach**	
Pessimistic HRH scale-up (1% Annual Growth)	Project an increase in HRH below historical trends, applying a constant 1% annual growth rate over 2025–2030.
Historical HRH scale-up (4.87% Annual Growth)	Project an increase in HRH based on historical growth trends from 2017 to 2024, maintaining a 4.87% annual growth (the average increase in HRH reported from 2017 to 2024) over 2025–2030.
Optimistic HRH scale-up (6% Annual Growth)	Project an accelerated increase in HRH above historical trends, applying a constant 6% annual growth rate over 2025–2030.
Consumables increased to 75th percentile	Align the availability of all consumables to the average availability seen at the 75th percentile of top-performing facilities, when the reported availability at a given facility is lower than the benchmark performer.
Consumables increased to HIV levels	Standardise the availability of all consumables by upgrading to match the higher average availability observed for HIV-related consumables, when the reported availability is lower than the HIV estimate from 2018.
Consumables increased to EPI levels	Standardise the availability of all consumables by upgrading to match the higher average availability observed for EPI-related consumables, when the reported availability is lower than the EPI estimate from 2018.
HSS Expansion Package	A comprehensive HSS package including:
	• Historical HRH Scale-up (4.87% Annual Growth)
	• Increase all consumables to 75th percentile
	Both implemented from 2025.
**HTM-focussed investment approach**	
HIV program scale-up	• Probability of PrEP for FSW after a negative HIV test: 50%
	• Probability of PrEP for AGYW after a negative HIV test: 10%
	• Probability of being retained on PrEP every 3 months: 75%
	• Probability of being retained on ART every 3 months: 100%
	• Probability of VMMC after HIV test for males: 25%
	• Annual testing rate among adults: 40%
	• Probability of HIV test at ANC or delivery: 95%
	• Probability of HIV test for newborns of HIV-diagnosed mothers: 95%
	• Probability of viral suppression if on ART: 95%
TB program scale-up	• Probability of a TB case being tested and referred for treatment: 90%
	• Probability of TB treatment success for drug-sensitive infection (adults): 90%
	• Probability of TB treatment success for MDR infection (all ages): 90%
	• Probability of TB treatment success for drug-sensitive infection (ages 0–4 years): 90%
	• Probability of TB treatment success for drug-sensitive infection (ages 5–14 years): 90%
	• First-line test for all presumptive TB cases: GeneXpert
	• Second-line test for all presumptive TB cases: Sputum smear microscopy
	• IPT coverage for PLHIV: 90%
	• IPT coverage for paediatric contacts of active TB cases: 90%
Malaria program scale-up	• Probability that a clinical or severe malaria case has a test: 100%
	• RDT testing rates among the general population: 10%
	• Probability of treatment success: 100%
	• Coverage of indoor residual spraying in the 11 highest-risk districts: 80%
	• Coverage of long-lasting insecticide-treated bednets in all districts: 70%
Joint HIV, TB, Malaria (HTM) programs scale-up	Includes all interventions listed in the above scenarios:
	• HIV program scale-up
	• TB program scale-up
	• Malaria program scale-up

All HTM-focussed program scale-up interventions are implemented from 2025.

*HRH* human resources for health, *EPI* expanded program on immunisation, *PrEP* pre-exposure prophylaxis, *FSW* female sex workers, *AGYW* adolescent girls and young women, *VMMC* voluntary medical male circumcision, *ANC* antenatal clinic, *ART* antiretroviral therapy, *MDR* multidrug resistant, *IPT* Isoniazid preventive therapy, *PLHIV* people living with HIV, *RDT* rapid diagnostic test.

The different investment approaches impose varying costs, making it essential to assess whether additional spending yields proportionate improvements in health outcomes, ensuring value for money. We assess this using Incremental cost-effectiveness ratios (ICERs) relative to a common *Baseline* scenario. ICERs are the ratio of the incremental cost of an investment to the DALYs averted through its implementation compared to the *Baseline* scenario or status quo. Additionally, we report the return on investment (ROI), a metric commonly used by donors^27^, which was estimated as the ratio of the net benefit (monetised value of health impact minus the incremental cost) to the incremental cost^27–29^.

## Results

With no expansion in health system capacity or disease program coverage above 2024 levels, we project 2.02 million deaths (95th percentile uncertainty interval [UI] 2.00–2.04 million) and 127.03 million DALYs (UI: 126.29–127.88 million) would occur between 2025 and 2035. Below, we present the health impact of the scenarios across the three investment approaches as well as their comparative cost-effectiveness and ROI. All results pertain to the 11-year period between 2025 and 2035.

### HTM-focussed investments

**Fig. 1:**
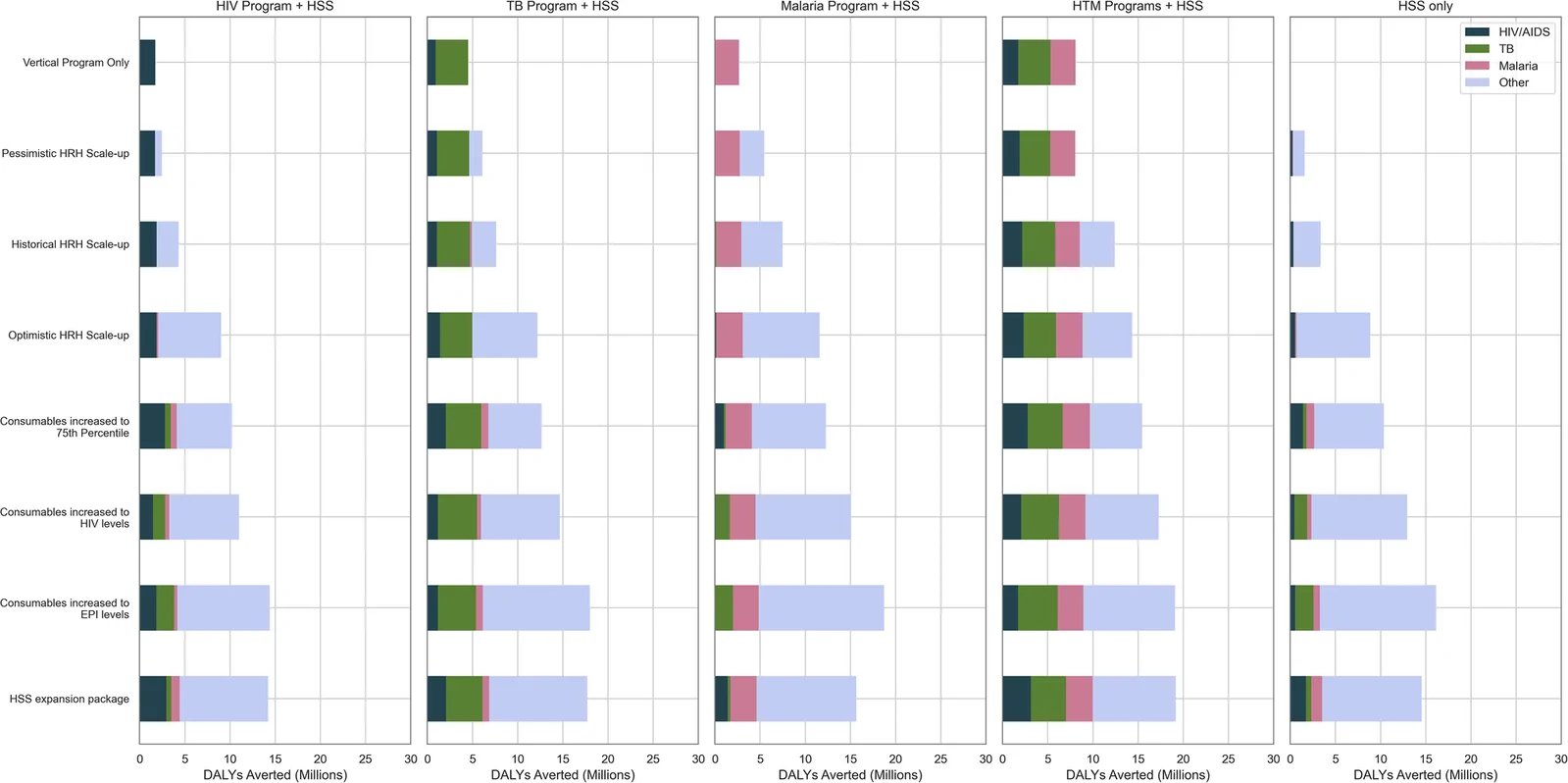
The breakdown of disability-adjusted life years (DALYs) averted (in millions) from 2025 to 2035, disaggregated by cause (HIV/AIDS, tuberculosis (TB), malaria, and “Other”—encompassing all remaining causes), for every intervention scenario. The first four panels show the HIV, TB, and malaria (HTM) programmes without and then with each component of health system strengthening (HSS), i.e., different levels of human resources for health (HRH) and consumable availability scale-up. The final panel shows the DALYs averted for HSS components alone. The comparison is against the *Baseline* scenario, in which health system capacity and service delivery remain fixed at 2024 levels throughout the projection period. Undiscounted values are presented. Values shown are the medians of five runs.

Focussed investments in HTM programs reduced disease burden but the scale of impact remained constrained by system bottlenecks that limited diagnosis, treatment availability, and service delivery overall (Fig. 1). *HTM Programs Scale-up Without HSS Expansion* averted 6.80 million (UI: 5.86–7.88 million) DALYs (4.98%, UI: 4.63–6.16%), reducing DALYs due to HIV/AIDS, TB and malaria by 27.09% (UI: 23.17–31.45%), 71.20% (UI: 63.02–74.69%) and 64.07% (UI: 58.41–64.96%) respectively (Table 2). This would reduce deaths due to HIV/AIDS, TB, and malaria by approximately 136,800. The coverage of treatment improved for HIV, TB, and malaria, from 90.37% (UI: 90.00–91.18%) in 2024 to 95.74% (UI: 95.42–96.12%) in 2035 for HIV, from 45.99% (UI: 41.84–51.17%) to 75.42% (UI: 72.75–82.05%) for TB, and 67.44% (UI: 66.39–67.60%) to 73.53% (UI: 73.48–74.60%) for malaria (Supplementary Fig. 1).

**Table 2:** Percentage of DALYs and deaths averted between 2025 and 2035 by scenario

Investment approach	Scenario	Percentage DALYs averted	Percentage deaths averted
**Health system strengthening (HSS)**	Pessimistic HRH scale-up	0.67 (−0.87 to 2.32)	0.60 (−1.25 to 1.43)
	Historical HRH scale-up	2.90 (2.38–4.22)	2.74 (0.86–3.43)
	Optimistic HRH scale-up	6.32 (5.34–7.50)	4.42 (3.68–5.93)
	Consumables increased to 75th Percentile	7.92 (6.44–8.84)	6.24 (5.29–6.34)
	Consumables increased to HIV levels	9.73 (8.51–10.50)	7.99 (7.05–8.83)
	Consumables increased to EPI levels	12.14 (11.03–13.11)	10.47 (8.66–10.83)
	**HSS expansion package**	**11.40 (10.46**–**12.08)**	**8.12 (7.98**–**9.41)**
**HTM-focussed**	HIV program scale-up without HSS expansion	0.44 (−0.02 to 1.37)	0.23 (−0.32 to 2.00)
	TB program scale-up without HSS expansion	3.16 (2.41–4.14)	3.85 (2.65–5.05)
	Malaria program scale-up without HSS expansion	1.94 (0.86–2.50)	1.49 (0.04–2.04)
	**HTM programs scale-up without HSS expansion**	**4.98 (4.63**–**6.16)**	**5.32 (4.85**–**6.58)**
**Combined**	HIV program scale-up with HSS expansion package	11.48 (10.14–11.79)	9.52 (7.79–9.66)
	TB program scale-up with HSS expansion package	14.17 (13.48–14.39)	12.16 (11.73–13.09)
	Malaria program scale-up with HSS expansion package	11.79 (11.17–13.36)	9.03 (8.35–10.72)
	**HTM programs scale-up with HSS expansion package**	**15.03 (14.46**–**15.94)**	**13.29 (13.04**–**14.87)**

Values show the median DALYs averted compared with *Baseline* across the five simulated runs for each scenario, with 95% uncertainty intervals in brackets defined as the 2.5th and 97.5th percentiles.

### Health systems strengthening investments

Between 2025 and 2035, HRH investments averted 0.67% (UI: −0.87 to 2.32%), 2.90% (UI: 2.38–4.22%) and 6.32% (UI: 5.34–7.50%) of DALYs under the *Pessimistic (1%), Historical (4.85%)*, and *Optimistic (6%) HRH* scale-up scenarios, respectively, compared to *Baseline*. Strengthening supply chains averted 7.92% (UI: 6.44–8.84%) of DALYS with *Consumables Increased to 75th Percentile*, 9.73% (UI: 8.51–10.50%) with *Consumables increased to HIV levels* and 12.14% (UI: 11.03–13.11%) with *Consumables increased to EPI levels*, with respiratory infections and diarrhoeal diseases benefiting most significantly. The *HSS expansion package* (combining *Historical HRH scale-up*, alongside *Consumables increased to 75th percentile*) averted 11.40% (UI: 10.46–12.08%) of DALYs. These investments contributed to substantial mortality reductions, reducing deaths due to HIV/AIDS (31,700), TB (9900), malaria (15,400) and other causes (126,600) over the same period. Baseline consumable availability varies substantially across programs and facility levels (Supplementary Fig. 2); consequently HSS improvements translate into non-linear improvements across multiple programs.

### Combined investments

Combined approaches, whereby HTM-focussed and health system strengthening investments are both applied, consistently outperformed all HTM-focussed approaches, reducing DALYs by 15.03% (UI: 14.46–15.94%) compared to a 4.98% reduction through vertical investments alone (Fig. 1). A substantial proportion of these gains (9.19 million DALYs [UI: 7.87–10.47 million]), were for all other causes (non-HTM), highlighting the broader health benefits of system-wide strengthening in addressing both communicable and non-communicable diseases. *HTM Programs Scale-up Without HSS Expansion* could avert 5.32% (UI: 4.85–6.58%) of deaths, whereas combined investments could avert 13.29% (UI: 13.04–14.87%) of deaths, an additional 158,200 lives saved. Furthermore, integrating HSS with HTM-focussed program expansion led to greater reductions in disease burden for each of the HIV, TB, and malaria programs than with only the HTM-focussed programs (Supplementary Fig. 1): additional reductions of 30,500 HIV/AIDS deaths, 8400 TB deaths and 3600 malaria deaths.

### Value for money

**Fig. 2:**
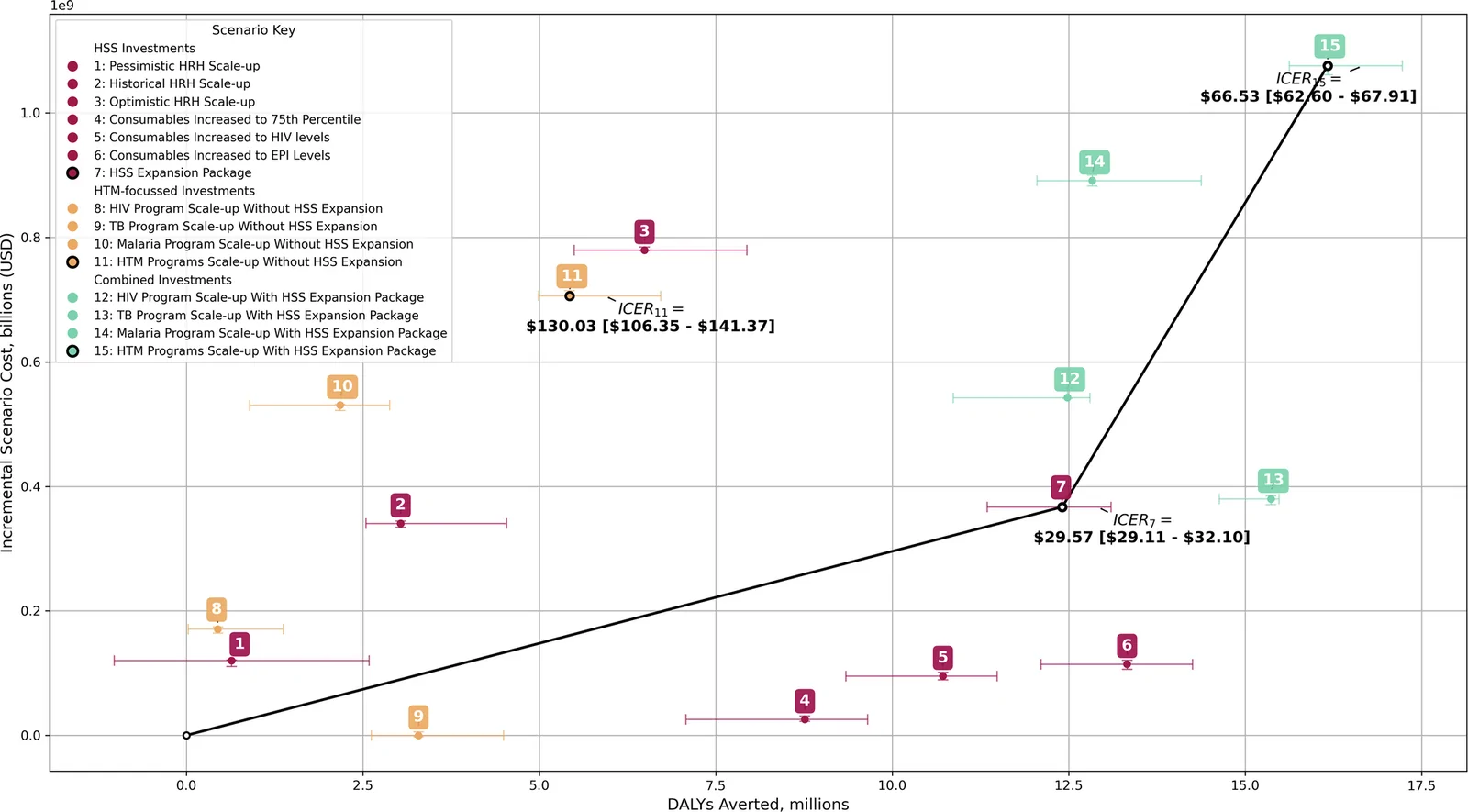
Disability-adjusted life years (DALYs) averted and incremental costs (compared to baseline) over the 11-year period (2025–2035) for all scenarios considered, categorised into health system strengthening (HSS)—including human resources for health (HRH) and consumable availability scale-up, HIV/Tuberculosis/Malaria (HTM)-focussed, and combined approaches. Cost and DALYs averted are both discounted annually at 3%. The origin of the plot represents the *Baseline* Scenario. The ratio of the incremental cost to DALYs averted represents the Incremental Cost-Effectiveness Ratio (ICER) of the scenarios, compared to the *Baseline* Scenario. The dark line represents the expansion path calculated within the restricted set of three alternative joint investment strategies (HSS Expansion Package, HTM programs scale-up without HSS Expansion, and HTM programs scale-up with HSS Expansion), which together form an exhaustive set for this comparison. Values shown are the median of five runs with uncertainty intervals derived from the 2.5th and 97.5th percentiles.

Overall, the HSS investments to strengthen consumables supply chains and the *HSS expansion package* (combining consumable availability improvement with HRH scale-up) yielded greater health benefits at lower costs compared to HTM-focussed approaches (Fig. 2). Moreover, the two approaches exhibited strong synergies, as evidenced by the incremental health gains when they are combined.

Focusing on the three joint investment scenarios (*HSS Expansion Package, HTM programs scale-up, and HTM programs scale-up with HSS Expansion)*, *HSS Expansion Package* strictly dominated *HTM programs scale-up without HSS*, delivering a greater health impact at a lower cost. It averted 12.41 (UI: 11.34–13.10) million discounted DALYs over the 11-year projection period, with an ICER of $29.57 (UI:$29.11–$32.10) per DALY averted, substantially lower than the *HTM programs scale-up*, which had an ICER of $130.03 (UI: $106.35–$141.37) per DALY averted.

Combining the two approaches resulted in even greater health gains (16.17, UI: 15.63–17.23 million DALYs averted), albeit at a greater cost ($1.08 billion, UI: $1.06–$1.08 billion), yielding an ICER of $66.53 (UI: $62.60–$67.91) per DALY averted. This represents a 49% reduction in cost per DALY averted relative to the HTM-focussed approach.

Among the three alternative joint investment scenarios*, HTM programs scale-up* is therefore the least efficient use of resources. It is dominated by the *HSS Expansion Package*—being more costly and less effective—and it is excluded by extended dominance when compared to the combined strategy—*HTM programs scale-up with HSS Expansion*. The expansion path (Fig. 2) selects the non-dominated scenarios that provide the greatest incremental health gains per dollar spent, following WHO-CHOICE guidelines^30^.

These estimates reflect only service-level costs, consistent with practical recommendations for cost-effectiveness analysis^31^, and exclude above service-level costs such as program management and outreach. Table 3 provides a detailed breakdown of ICERs for all scenarios compared with the *Baseline*. The relative cost-effectiveness of scenarios remains qualitatively the same under alternative discount rate assumptions (Supplementary Figs. 4 and 5).

**Table 3:** Incremental cost-effectiveness ratio by scenario

Investment approach	Scenario	ICER (2023 USD) *(in comparison with the baseline scenario which assumes that health system capacity remains at 2024 levels)*
**Health system strengthening (HSS)**	Pessimistic HRH scale-up	$186.69 [$46.71–Dominated]
	Historical HRH scale-up	$112.03 [$75.90–$131.35]
	Optimistic HRH scale-up	$120.05 [$98.79–$141.74]
	Consumables increased to 75th percentile	$3.18 [$2.92–$3.22]
	Consumables increased to HIV levels	$8.88 [$8.79–$9.52]
	Consumables increased to EPI levels	$8.58 [$8.48–$8.77]
	**HSS expansion package**	**$29.57 [$29.11**–**$32.10]**
**HTM-focussed**	HIV program scale-up without HSS expansion	$382.18 [$126.71–$5714.48]
	TB program scale-up without HSS expansion	Dominant [Dominant–$1.29]
	Malaria program scale-up without HSS expansion	$243.32 [$184.74–$582.76]
	**HTM programs scale-up without HSS expansion**	**$130.03 [$106.35**–**$141.37]**
**Combined**	HIV program scale-up with HSS expansion package	$43.54 [$43.47–$49.85]
	TB program scale-up with HSS expansion package	$24.85 [$24.70–$25.36]
	Malaria program scale-up with HSS expansion package	$69.43 [$62.59–$73.26]
	**HTM programs scale-up with HSS expansion package**	**$66.53 [$62.60**–**$67.91]**

ICER estimates only include service-level costs and assume zero above service level costs. Values show the median ICERs with uncertainty intervals derived from the 2.5th and 97.5th percentiles. All costs are expressed in 2023 US dollar terms.

Further, recognising the need to account for limited fiscal space when sequencing investments, we decompose the broader investment strategies into five independent constituent options—*HIV program scale-up*, *TB program scale-up*, *Malaria program scale-up*, C*onsumables increased to the 75th percentile*, and *Historical HRH scale-up*—and evaluate all 31 possible combinations of these against the baseline. The resulting expansion path comprises: (1) *TB program scale-up*; (2) *TB program scale-up with consumables increased to the 75th percentile*; (3) *TB program scale-up with HSS Expansion*; and (4) *TB and malaria program scale-up with HSS Expansion*. These scenarios represent progressively higher investment levels that deliver the greatest incremental health benefits per dollar spent (Supplementary Fig. 3).

### Return on investment

**Fig. 3:**
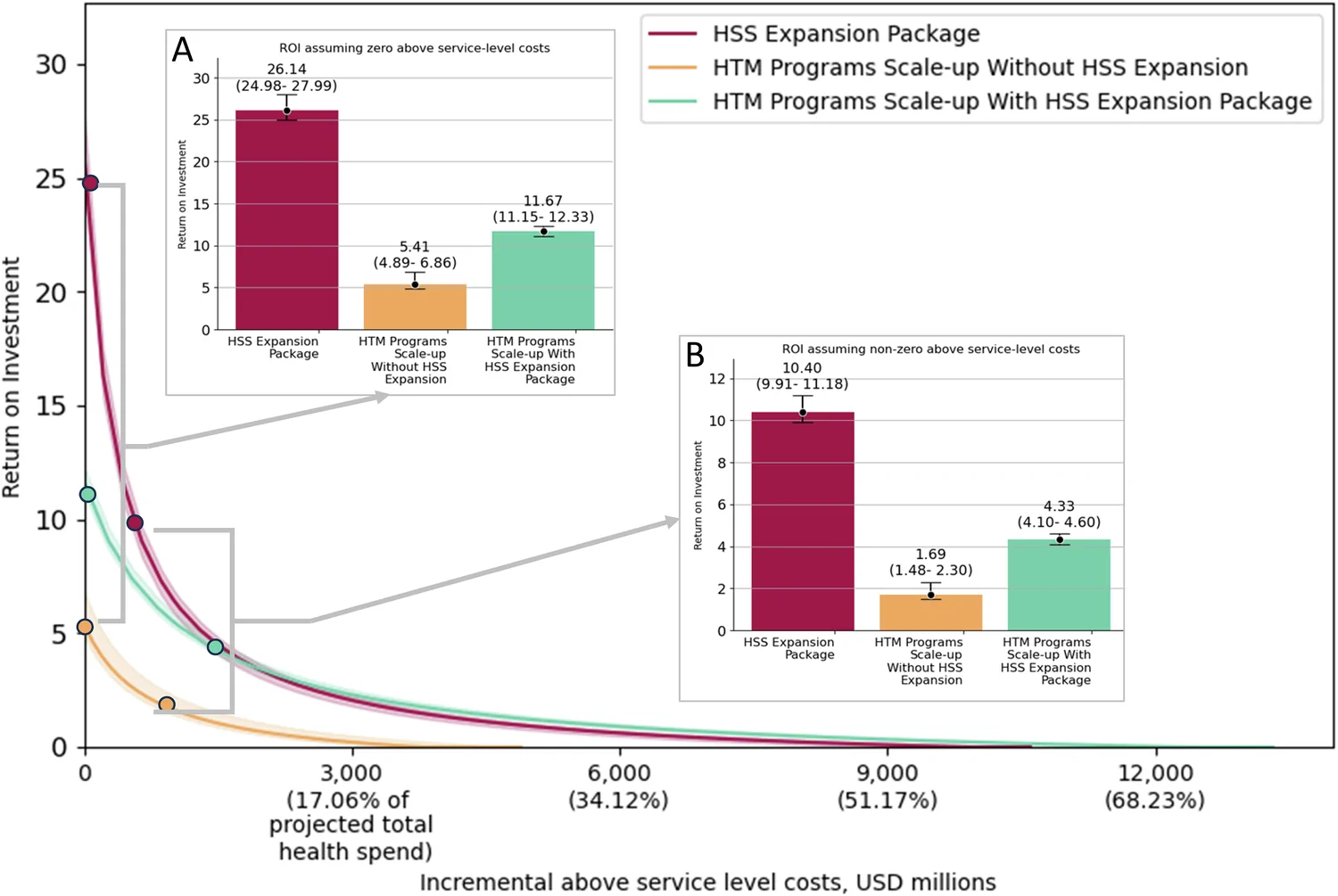
Return on investment across a range of hypothetical incremental above service-level costs. Return on investment (ROI) is shown for health system strengthening (HSS Expansion Package), scale-up of HIV, tuberculosis and malaria programmes without HSS expansion (HTM Programs Scale-up Without HSS Expansion), and HTM programme scale-up with HSS expansion (HTM Programs Scale-up With HSS Expansion Package), across a range of hypothetical incremental above-service-level costs. Inset bar plots show ROI under two specific assumptions: (**A**) zero above-service-level costs and (**B**) above-service-level costs equivalent to 138% of service-level costs. Values are medians of five runs, with uncertainty intervals representing the 2.5th and 97.5th percentiles.

With a value of a statistical life year (VSLY) of $834^32^ and assuming no incremental above service-level costs, the estimated ROI was 26.14 (UI: 24.98–27.99) for the *HSS Expansion Package*, 11.67 (UI: 11.15–12.33) for the *HTM programs scale-up with HSS Expansion Package*, and 5.41 (UI: 4.89–6.86) for *HTM programs scale-up without HSS Expansion Package* (Table 4 and Fig. 3). While all three scenarios delivered a high ROI, the HSS expansion scenario had a median ROI 4.8 times that of the HTM-focussed scenario and 2.2 times the combined scenario. Assuming incremental above service-level costs equal to 138% of service-level cost (based on estimates from ref. ^33^) of each scenario, the ROI of the HSS, combined, and HTM-focussed scenarios dropped to 10.40 (UI: 9.91–11.18), 4.33 (UI: 4.10–4.60), and 1.69 (UI: 1.48–2.30) respectively, preserving the same relative ordering.

**Table 4:** Return on investment by scenario

Scenario	Monetised health benefits ($, billion)	Service-level costs ($, million)	Above Service-level costs ($, million) *(assumed to be equal to 138% of service-level costs)*	ROI (assuming zero above service-level costs)	ROI (assuming non-zero above service-level costs)
Pessimistic HRH scale-up	$0.54 [$0.00–$2.16]	$119.97 [$110.59–$121.04]	$165.56 [$152.62–$167.04]	3.52 [−1.0 to 16.87]	0.9 [−1.0 to 6.51]
Historical HRH scale-up	$2.53 [$2.12–$3.78]	$340.27 [$334.23–$344.35]	$469.57 [$461.23–$475.20]	6.47 [5.35–9.99]	2.14 [1.67–3.62]
Optimistic HRH scale-up	$5.41 [$4.58–$6.62]	$779.35 [$778.99–$784.63]	$1,075.51 [$1,075.01–$1,082.79]	5.95 [4.88–7.46]	1.92 [1.47–2.56]
Consumables increased to 75th percentile	$7.31 [$5.90–$8.05]	$25.59 [$22.54–$31.12]	$35.31 [$31.10–$42.95]	248.72 [193.19–353.97]	103.93 [80.59–148.15]
Consumables increased to HIV levels	$8.94 [$7.79–$9.58]	$95.20 [$89.01–$100.96]	$131.37 [$122.83–$139.33]	93.36 [80.32–105.92]	38.65 [33.17–43.93]
Consumables increased to EPI Levels	$11.12 [$10.10–$11.89]	$114.35 [$106.27–$120.84]	$157.80 [$146.65–$166.76]	97.97 [87.34–105.04]	40.59 [36.12–43.55]
**HSS expansion package**	**$10.35 [$9.46**–**$10.93]**	**$366.95 [$364.19**–**$381.44]**	**$506.39 [$502.58**–**$526.39]**	**26.14 [24.98**–**27.99]**	**10.4 [9.91**–**11.18]**
HIV program scale-up without HSS expansion	$0.37 [$0.03–$1.14]	$170.46 [$164.08–$173.96]	$235.24 [$226.44–$240.07]	1.14 [−0.83 to 5.65]	−0.1 [−0.93 to 1.79]
TB program scale-up without HSS expansion	$2.74 [$2.19–$3.75]	$−0.33 [$−2.22 to $5.78]	$−0.46 [$−3.06 to $7.97]	953.22 [648.7–9029.62]	401.09 [271.98–3794.54]
Malaria program scale-up without HSS expansion	$1.82 [$0.75–$2.40]	$530.42 [$521.81–$532.04]	$731.98 [$720.10–$734.22]	2.43 [0.43–3.52]	0.44 [−0.4 to 0.9]
**HTM programs scale-up without HSS expansion**	**$4.53 [$4.16**–**$5.60]**	**$706.15 [$705.04**–**$714.71]**	**$974.49 [$972.96**–**$986.30]**	**5.41 [4.89**–**6.86]**	**1.69 [1.48**–**2.3]**
HIV program scale-up with HSS expansion package	$10.41 [$9.06–$10.68]	$542.65 [$541.61–$557.39]	$748.85 [$747.42–$769.19]	17.84 [15.7–18.22]	6.92 [6.02–7.07]
TB program scale-up with HSS expansion package	$12.82 [$12.20–$12.91]	$379.67 [$371.05–$384.69]	$523.95 [$512.05–$530.88]	33.01 [31.1–33.69]	13.29 [12.49–13.57]
Malaria program scale-up with HSS expansion package	$10.70 [$10.05–$11.99]	$891.22 [$882.74–$899.92]	$1,229.89 [$1,218.19–$1,241.89]	11.01 [10.3–12.57]	4.05 [3.75–4.7]
**HTM programs scale-up with HSS expansion package**	**$13.48 [$13.03**–**$14.37]**	**$1,075.65 [$1,061.28**–**$1,078.40]**	**$1,484.39 [$1,464.56**–**$1,488.20]**	**11.67 [11.15**–**12.33]**	**4.33 [4.1**–**4.6]**

Return on investment values assume a Value of Statistical Life Year = $834. Values show the median ICERs with uncertainty intervals derived from the 2.5th and 97.5th percentiles. All costs are expressed in 2023 US dollar terms.

These results indicate that *HSS Expansion Package* consistently yields the highest ROI, regardless of assumptions about above service-level costs. While combined investments in HTM and HSS increase health impact, they deliver lower returns per dollar invested than HSS on its own.

These findings remained consistent under alternate assumptions of discount rates and VSLY (Supplementary Figs. 6 and 7 and Supplementary Tables 4–7).

## Discussion

This study quantifies for the first time the health impact of supply chain strengthening and HRH scale-up, showing that integrating aspects of HSS with disease-specific program scale-up delivers substantially greater health benefits and value for money than standalone HTM-focussed interventions. The combined approach averted three times as many DALYs as HTM-focussed programs alone, due to expansion of the health workforce and improved availability of essential consumables. It also proved more cost-effective, with an ICER of $67 per DALY averted against the baseline, compared to HTM program expansion (ICER $130/DALY averted). This aligns with earlier studies showing the importance of addressing system constraints to maximise the effectiveness of disease-specific interventions^1,7,12^.

Different HTM components are limited by different constraints, making the marginal return to HSS inherently intervention-specific. For supply-constrained services (e.g., TB diagnosis reliant on GeneXpert or chest x-ray), additional staff capacity and more reliable stocks yield larger gains. For demand-sensitive services (e.g., pre-exposure prophylaxis (PrEP) for HIV), demand-generation is the binding constraint, with smaller pay-offs from supply-side HSS alone. Although the analysis focuses on Malawi, the mechanisms identified are relevant across other LMICs. The balance between disease-focussed and HSS investments is expected to differ with the maturity and capacity of individual health systems. In less-developed systems, HTM scale-up can remain highly effective until foundational service delivery capacities are in place^1,11,12,17,18,34^; as systems strengthen, the relative value of broader HSS investments increases^35,36^. Future comparative analyses could help quantify this transition and clarify how system development shapes optimal investment strategies.

While our model accounts for real-world constraints such as workforce shortages and stockouts, uncertainties in epidemiological parameters and cost estimates may affect projection precision. We incorporate stochastic variation in key processes, reporting uncertainty across aggregated runs, and consider a number of alternative assumptions through the sensitivity analysis. Despite positive health gains for HIV-specific outcomes, overall DALYs and deaths did not significantly improve under the *HIV Program scale-up* without accompanying health system strengthening (0.44% [−0.02 to 1.37] DALYs averted and 0.23% [−0.32 to 2.00] of deaths averted). In this model, resources are finite and shared across programs; increasing provision for HIV necessarily reduces the capacity available for other conditions, displacing or delaying care. This reflects the high baseline performance of the HIV care cascade and the modest short-term impact of expanding prevention activities, which, while resource-intensive, yield limited reductions in DALYs within the timeframe of this analysis. This finding, in which disease-focused investments can negatively affect non-target services, is consistent with empirical evidence that vertical programs have sometimes diverted resources away from primary care^37^. In Malawi, bilateral or NGO programs provide services outside Ministry of Health's (MoH) administrative control, and these workers and patient volumes may be incompletely enumerated in MoH HRH and service statistics^38^. For our MoH-centred investment strategy analysis, using the MoH roster is appropriate; however, if substantial off-platform capacity exists, our estimates of crowd-out within MoH facilities may be upper bounds. Conversely, where NGO staff are seconded to MoH facilities but not fully recorded, effective public capacity may be under-counted. We therefore interpret displacement effects as public-platform effects, and caution against extrapolating to the entire mixed health system without additional data on parallel platforms.

The ICER and ROI estimates reflect both direct costs of expanding health system inputs and attributable indirect costs under HTM-focussed and HSS scenarios. However, they do not account for potential additional administrative, regulatory, and implementation costs (above service-level costs^33^) that may arise when operationalizing these scenarios, and so we present ROI estimates across a range of hypothetical above service-level costs over which the ROI remains positive. This is particularly important in the case of modelling improvements in consumable availability, for which our incremental costs include the cost of additional consumables stocked (along with proportional supply chain costs) and not the broader operational interventions that may be needed to enable these improvements (e.g., logistics systems, expanded storage or distribution capacity)^24^. Additionally, our costing method does not systematically account for incremental equipment costs relative to the baseline^39^. This is because the quantity of medical equipment is assumed to match the levels prioritized in the national strategic plan across all scenarios, due to a lack of data on the throughput of each equipment type.

In the absence of robust estimates of such expenditures tied to specific HTM-focussed and HSS expansion options, we present ROI results under the assumptions of zero above service-level costs or a 138% overhead, serving as lower and upper bounds for interpretation. The fact that the health systems strengthening approach outperforms both the HTM-focussed and combined strategies across such a wide range of above service-level cost assumptions lends confidence to the robustness of our conclusions.

While our model accounts for several important constraints within the health system, there remain additional factors such as weak information systems, inadequate infrastructure, and governance challenges that could influence both the effectiveness and wider applicability of HSS efforts. Although we model the expansion of the health workforce and improvements in supply chains as mechanisms to increase service coverage, it is important to recognise that the success of these measures also depends heavily on factors such as the quality of training, staff retention, and the equitable distribution of personnel across different regions and levels of care^40^. If domestic financing does not replace declining external support, near-term capacity could contract, implying our *Baseline* comparisons may overstate achievable coverage relative to a contracting trajectory^23^. In this framework, some benefits accumulate over time via transmission, disease progression and repeated care use, but we do not represent gradual improvements in productivity or quality that might arise from enhanced training or supervision. As a result, the timing of gains may appear earlier than would appear in practice, whereas longer-term improvements that accrue slowly through sustained quality enhancement may be understated. Although integrating HTM-focussed programs with broader system-strengthening strategies can create synergies, limited resources inevitably generate trade-offs. In the simulated scenarios, services are prioritised with emergency care first, followed by HIV, tuberculosis, malaria, and then other services. Even with targeted HRH increases, staff may be diverted from other essential areas. Careful, context-specific analysis of resource distribution and the consequences of prioritisation decisions is therefore critical to maximise impact and ensure sustainability.

This evaluation provides evidence to inform policy dialogues with government and partners that argues for increased investment in health system strengthening. The analysis shows that expanding capacity through which services are delivered materially changes the attributable population health gains and alters the efficiency profile of subsequent program investments. By making these trade-offs explicit, the study equips decision-makers with an empirically grounded means to examine the consequences of reallocating resources across system inputs and program activities. As health sectors in many countries face rising demands within limited fiscal space, such analytically grounded assessments can strengthen the evidence base for strategic planning and improve the robustness of long-term investment decisions.

## Methods

### Ethical approval

The Thanzi La Mawa project received ethical approval from the College of Medicine, Malawi Research Ethics Committee (P.09/23-0297) for the use of publicly accessible and anonymised secondary data. No data requiring individual informed consent were used.

### Study design and model overview

The modelling analysis was performed using the TLO model, an individual-based simulation, to estimate the health impact of investments in HRH, consumables, and disease-specific programs in Malawi^25^. The model represents interactions between individuals and the health system, capturing a wide spectrum of conditions (e.g., communicable diseases, non-communicable diseases, maternal and newborn health) and incorporating demographic, behavioural, and epidemiological processes. This dynamic framework captures the population-level effects of health interventions, including indirect (herd) effects where appropriate. For infectious diseases such as HIV, tuberculosis, and malaria, the model explicitly represents person-to-person transmission dynamics, with transmission probabilities varying as a function of intervention coverage and population prevalence. As such, increases in intervention coverage—for example, in vector control, ART scale-up, or TB case detection—lead to reductions in incidence not only in those directly receiving the intervention but also in the wider population, through decreased onward transmission. Detailed model structure and calibration procedures are described elsewhere^25,41^.

Healthcare interactions are modelled as discrete, time-ordered events that capture the full complexity of service delivery within a constrained health system. Each interaction is operationalised through a detailed “appointment footprint” that quantifies the clinical, nursing, pharmacy, and radiography resources (personnel, patient-facing time, consumables and equipment) required for a given service. An appointment footprint defines the resources for a single completed contact: (i) cadre-specific minutes, (ii) any diagnostics required, and (iii) consumables/medicines/equipment. This approach not only defines the resource use associated with routine care but also dynamically accounts for disruptions due to consumable or health workforce shortages; when appointments cannot run, protocol-specific repeat appointments and/or referral pathways to higher-level facilities will occur.

### Health system structure

Malawi’s health system is structured into three levels: primary, secondary, and tertiary care, each serving distinct roles for healthcare delivery. Primary care encompasses community-based outreach, health posts, dispensaries, urban health centres, and primary health centres (collectively referred to as level 0 or 1a in the TLO model), and rural or community hospitals (referred to as level 1b)^22^. These facilities provide outpatient, maternity, and antenatal services. Secondary care is delivered through 26 district hospitals, each offering services available at the primary level, supplemented by additional capabilities such as X-ray imaging, ambulance services, operating theatres, and laboratory facilities. Tertiary care is provided by four central hospitals located in major urban areas, offering specialised services and advanced medical care.

The MoH oversees national policy implementation, with service delivery managed at the district level through District Health Management Teams. Health facilities across all levels are required to maintain records of medical consumables through an electronic stock management tool (OpenLMIS system)^42^, which collects and aggregates data on drug stock levels, usage rates, and stockouts.

The TLO model incorporates detailed data on the healthcare workforce in Malawi to assess the alignment between healthcare service demand and workforce capacity^22^. This integration is achieved by formalising how the time of different healthcare worker cadres—including clinicians, nurses and midwives, pharmacists, and other essential staff—is allocated to healthcare services. The model utilises data from routine health management information systems and official records, incorporating only filled positions (excluding established but vacant posts) to provide a comprehensive view of workforce capacity. We assume that the productivity of healthcare workers to deliver healthcare services remains fixed over the duration of the simulation.

The human resources data provides detailed estimates of patient-facing time available per day for different health worker cadres across each facility level in Malawi^21^. These estimates provide an empirically grounded estimate of health worker availability and were used to assess the capacity of the health system to deliver services and model the impact of workforce constraints on health outcomes. The model represents service delivery through government and CHAM facilities, but excludes capacity from other private providers. Human resources are parameterised from MoH records of funded and filled posts, and service capacity is generated by allocating patient-facing time from these cadres across services. Parallel, donor-financed NGO or private delivery platforms are not modelled as additional capacity unless operating through MoH facilities and captured in MoH routine data. COVID-19-related disruptions are not modelled here. Baseline HRH and supply inputs reflect a non-pandemic steady state, representing current conditions rather than acute 2020–2022 disruption.

Appointment durations for healthcare services in Malawi are determined based on estimates of patient-facing minutes required for 49 different categories of appointments^22^, considering the average duration required for consultations, diagnostics, and treatment. These estimates help assess the capacity of healthcare workers and the number of patients they can serve within a given time frame, thus informing the modelled constraints on healthcare delivery^43^.

### HIV, tuberculosis and malaria (HTM) programs

The HIV program delivers a comprehensive suite of interventions, including voluntary testing and counselling, initiation of first-line ART, and targeted preventive measures such as voluntary medical male circumcision and PrEP for select high-risk populations. Treatment protocols are lifelong and aimed at achieving viral suppression; poor adherence and/or stockouts in consumables can lead to individuals defaulting from treatment and re-initiating at a later stage. The TB program employs established diagnostic modalities (principally sputum smear microscopy and GeneXpert assays) to identify active TB cases, which are then managed using standardised treatment regimens for both drug-sensitive and multidrug-resistant TB, supplemented by preventive therapy for identified paediatric contacts and people living with HIV. The malaria program is based on the widespread deployment of rapid diagnostic tests (RDTs) for case identification, followed by treatment in accordance with national guidelines for uncomplicated and severe malaria, and is supported by routine vector control measures, including the distribution of insecticide-treated bednets and periodic indoor residual spraying in high-risk districts.

### Investment approaches

Three investment approaches were investigated over the period 2025–2035, with multiple scenarios considered under each (see Table 1 for details):

1. HSS investment approach: This involves investments in health system strengthening, focusing on expansion of the healthcare workforce and improving the availability of essential medical consumables with improved healthcare-seeking behaviour in response to health system expansion.
2. HTM-focussed approach: Targeted expansions in HIV, TB, and malaria programs within existing health system constraints, including improvements in prevention, diagnostics, and treatment coverage for each disease. Demand for HTM services is increased in response to increased program coverage.
3. Combined approach: This approach integrates the HTM-focussed and HSS approaches, leveraging system-wide improvements to maximise the effectiveness and reach of disease-specific interventions while addressing broader health system limitations.

We implement the three approaches as program directives that determine where marginal MoH funds are placed and how service delivery is structured. HTM-focussed scenarios scale disease-specific interventions (HIV, TB, malaria) within prevailing system constraints. HSS scenarios expand cross-cutting enablers (HRH and consumable availability) that relax those constraints across services. Combined scenarios jointly expand HTM programs and the HSS package. The model translates these directives into changes in human resource capacity (appointments, staff time), likelihood of consumables being available and coverage in MoH facilities and then simulates the health and economic outcomes. A *Baseline* scenario holds MoH service capacity and HTM coverage targets fixed at their 2024 levels for 2025–2035.

#### Increases in HRH

To model the increase in healthcare staff over time, we used a curve-fitting approach to derive a scaling factor that reflects the actual growth in staff numbers reported by Malawi MoH from 2017 to 2024 (Supplementary Fig. 2a). The scaling factor for each year was estimated using an exponential function:

1 $${Scale}\,{up}(y)\,=\,\exp (\beta \,{\cdot }\,(y-{y}_{{start}}))$$

where *y* represents the year, *y*_*start*_ is the starting year (2017), and β is a parameter defining the rate of growth. This function was fitted to historical data on HRH (using SciPy, Python v3.8) which estimates the best-fitting values for β. The data were normalised to the baseline 2017 staff number. The fitted function generated scaling factors for the years 2017 to 2024. For the years 2025–2030, the scale-up pattern was extended linearly, after which staff counts remained fixed. The average growth rate for the HRH Scale-up scenario between 2017 and 2030 was calculated using the compound annual growth rate formula:

2 $${{CAGR}=\left(\frac{2030\,{staff}{count}}{2017\,{staff}{count}}\right)}^{\frac{1}{{number}{of}{years}}}-1$$

giving an average annual growth rate 4.87% from 2017 to 2030. This represents the *Historical HRH Scale-up* scenario.

Two further generalised scenarios for HRH scale-up were considered, applying uniform annual growth rates of 6% (*Optimistic HRH Scale-up*) and 1% (*Pessimistic HRH Scale-up*) across all cadres and facility levels. Beyond 2030, we assume that workforce levels would stabilise as part of a longer-term planning framework. While the model runs through to 2035, the 2025–2030 scale-up period allows for a realistic projection of the impact of HRH investments within the typical planning and budgeting cycles used in health systems. The choice to keep workforce levels constant after 2030 is also aligned with the aim of assessing the medium-term impact of scaling up HRH under varying growth scenarios, without introducing excessive uncertainty about future workforce policies or interventions beyond the model’s timeframe.

#### Consumables availability

Three realistic scenarios were considered to modify consumable availability in Malawi at facility levels 1a and 1b, the levels where the majority of health services are delivered and where the issue of consumable stock-outs is most acute^24^. These scenarios involved adjustments to the availability of drugs, diagnostics, and other non-therapeutic supplies (e.g., gloves, syringes):

1. *Consumables Increased to 75th Percentile*: For each consumable, the availability at facility levels 1a and 1b was increased to match the benchmark facility (within the corresponding level) at the 75th percentile of availability. The final availability for each consumable was set to the higher value between its benchmark facility and the current reported availability.
2. *Consumables increased to HIV Levels*: Consumable availability was matched by facility level to the average availability of all consumables supplied through HIV program supply chains. This includes diagnostics, drugs, and HIV-specific medical items. For each consumable, availability was set to the greater of the current reported availability or the HIV program supply availability.
3. *Consumables increased to EPI Levels*: Similarly, consumable availability was matched by facility level to the average availability of all consumables supplied through the Expanded Program on Immunization (EPI) program supply chains, which included vaccines and vaccine-specific medical items (e.g., syringes, safe disposal boxes). For each consumable, availability was set to the greater of the current reported availability or the EPI program supply availability. 

Supplementary Fig. 2b presents the average availability of consumables by disease/public health program across these scenarios.

#### Constraints in health system supply

In the TLO model, both HRH time and essential commodities are explicitly constrained. Each health worker cadre has a finite number of patient-facing hours available per day, which are allocated across all service demands within their district according to defined task profiles. We define a probability that each consumable (e.g., diagnostic tests, medicines, preventive commodities) will be available at different facility levels in different districts based on empirical estimates of the supply chain performance. Scaling up services for HIV, TB, or malaria reduces the availability of staff hours for other conditions, particularly where cadres are shared across programs. This mechanism captures one channel through which program-focused investments can negatively affect care for non-target diseases by displacing or delaying service provision elsewhere in the system.

### Economic analysis

A mixed-method costing approach was adopted to cost the health system resources estimated to be used under all scenarios^39^. Specifically, we applied bottom-up micro-costing to resources that are directly attributable to service delivery and apportionable by patient, such as medical consumables dispensed and health worker salaries. For higher-level costs, such as the distribution and warehousing of medical consumables (supply chain), monitoring and supervision of health workers, and other overheads, we adopted a top-down approach. The bottom-up approach uses detailed estimates of units of each resource used for healthcare delivery. The top-down approach relies on empirical estimates of the proportion of resource costs directly used for service delivery that are allocated to such overheads within Malawi. The following costs were included:

1. Medical consumables—including the cost of the quantity of consumables dispensed, quantity expected to be wasted, and supply chain overheads.
2. Human resources for health—including the cost of standard salaries, cost of in-service training and supervision overheads of the total workforce, cost of pre-service training to overcome regular health worker attrition as well as expand the workforce in certain scenarios
3. Medical equipment—including the annuitized replacement cost of equipment over their lifespans as well as the cost of repair, maintenance and spare parts.
4. Facility operating costs—including utility bills, maintenance, administrative staff, and other overheads.
5. Malaria scale-up costs—The full implementation cost of scaling up insecticide-treated bednet distribution and indoor residual spraying (IRS) was estimated using figures from Stelmach et al.^44^, updated with the latest consumable costs from Malawi. The cost was estimated at $1.4 per person covered by a bednet per year (assuming a 3-year lifespan and an average of 1.8 people per bednet) and $3.5 per person covered by IRS.

All costs are estimated from a healthcare provider perspective and are expressed in 2023 US dollar terms.

Health benefits were quantified using DALYs averted. DALYs were computed as the sum of Years of Life Lost (YLL) due to premature mortality and Years Lived with Disability^45^. ICERs were estimated as the ratio of incremental costs and incremental health benefit, using the following standard formula –

3 $$\frac{{{Service}\,{level}\,{cost}}_{{alternative}\,{scenario}}\,-\,{{Service}\,{level}\,{cost}}_{{baseline}}}{{{{DALYs}{incurred}}_{{baseline}}-{DALYs}{incurred}}_{{alternative}\,{scenario}}}$$

These costs represent “service-level costs” which are used in standard cost-effectiveness analyses^31^. Supplementary Table 1 presents a breakdown of incremental costs for each scenario.

To estimate ROI, health benefits measured in terms of DALYs averted were monetised using an estimate of the VSLY in Malawi. For our main analysis, we used the VSLY estimate of $834, which has previously been used by the Government of Malawi^32^. The ROI was then estimated as follows^27–29^

4 $$\frac{{Monetised}\,{health}\,{benefits}\,-\,{Incremental}\,{service}\,{level}\,{cost}\,}{{Incremental}\,{service}\,{level}\,{cost}}$$

In addition to service-level costs, healthcare programs often incur substantial “above service-level costs”, such as startup expenses, program management, outreach, technical assistance, and broader administrative functions. For example, Opuni et al.^33^ estimated that above service-level costs accounted for 58% of the total cost of the LINKAGES program for key populations affected by HIV in Malawi, or 138% of service-level costs^33^. Given the absence of data on these overheads for the scenarios modelled in this study, we report ROI estimates across a range of hypothetical values for these unknown above service-level costs. To assess the robustness of our conclusions, we focus our interpretation on the range of plausible above service-level costs between 0 and 138% of service-level costs. This range reflects both optimistic assumptions (i.e., minimal incremental above-service overheads associated with implementing each scenario) and empirical evidence from a targeted program in Malawi that is likely to have incurred relatively high above service-level cost, higher than those expected under our integrated scenarios, which are designed to leverage and strengthen the existing health system infrastructure.

To incorporate above service-level costs, the formula to calculate ROI is updated as follows –

5 $$\frac{{Monetised}\,{health}\,{benefits}\,-\,({Incremental}\,{service}\,{level}\,{cost}\,+\,{Incremental}\,{above}\,{service}\,{level}\,{cost})\,}{{Incremental}\,{service}\,{level}\,{cost}\,+\,{Incremental}\,{above}\,{service}\,{level}\,{cost}}$$

To aid the interpretation of different levels of above service-level costs, we express them as a percentage of projected total health spending under the *Baseline* scenario. This is estimated by multiplying projected estimates of per capita health spending from Dieleman et al. ^46^ between 2025 and 2035 with the population projected over this period by the TLO model. Supplementary Table 2 presents the annual projected health spending between 2025 and 2035.

To avoid misleading results for scenarios where the incremental cost is negative (i.e., the scenario results in net cost savings relative to the baseline), we use the absolute value of the incremental cost in the denominator for the ROI formula.

Further, we construct a cost-effectiveness frontier across scenarios and report an expansion path that sequences options with the highest health gain per additional dollar, following WHO-CHOICE guidelines^30^. The method is described in further detail in Supplementary material section B.1.

### Model simulations and calibration

A representative cohort (100,000 simulated individuals, i.e., a model to actual population size ratio of 1:147) of the Malawian population was simulated from 2010, assigning demographic, lifestyle, and health characteristics according to reported survey, census, epidemiological and health system usage data. The model is executed in Python programming language 3.8^47^.

To account for stochastic variability, each scenario was simulated five times, and results were summarised using the median and 95% uncertainty intervals (derived from 2.5th and 97.5th percentiles). Comparisons across scenarios were performed using pairwise run comparisons, so that each scenario shared an identical underlying stochastic trajectory within each draw; this allowed differences in outcomes to be attributed to the intervention rather than random variation. Stochastic variation arose from individual-level heterogeneity in disease progression, transmission dynamics, and healthcare interactions. The model was calibrated against historical epidemiological data, national databases of healthcare service delivery, including records of patient appointments, consumable stockouts, and health workforce deployment, as well as independent estimates from sources such as UNAIDS and WHO^25^. DALYs are assigned (“stacked”) at the date of death, such that all future YLL attributable to that death are accrued on that date.

### Sensitivity analysis

To assess the robustness of the economic conclusions, sensitivity analyses were conducted on key parameters influencing the ICERs and ROI estimates.

#### Discount rate

Health and economic outcomes were evaluated under multiple discounting assumptions to account for different time preferences regarding the value of costs and benefits, particularly relevant for interventions whose benefits accrue over the long term. Consistent with standard economic evaluations, the primary analysis applied a 3% annual discount rate to both health and costs^48^. Following WHO-CHOICE methods, results were additionally reported with zero discounting of health outcomes and 3% discounting of costs^30^. Finally, recognising the potential for higher growth rates in low-income countries, we reported results assuming a 5% discount rate for both health and costs^48^. Estimating ICER and ROI under varying discount rate assumptions enabled us to assess the robustness of our findings to different societal time preferences regarding the value of future costs and health benefits.

#### Value of a statistical life year (VSLY)

Given the inherent uncertainty in assigning a monetary value to health benefits, alternative VSLY estimates were considered. The main analysis assumed a VSLY of USD $834, reflecting a prior valuation used by the Government of Malawi^32^. For our sensitivity analysis, we re-estimated the VSLY in 2025 following ref. ^49^, by dividing the value of a statistical life (VSL) in Malawi by the conditional life expectancy at the age equal to half of the life expectancy at birth. The VSL for Malawi was estimated using the following formula^49^,

6 $${{{{\rm{VSL}}}}}_{{{{\rm{Malawi}}}}}={{{{\rm{VSL}}}}}_{{{{\rm{USA}}}}}*{({{{{\rm{GNI}}}}}_{{{{\rm{pp}}}}{{{\rm{Malawi}}}}}/{{{{\rm{GNI}}}}}_{{{{\rm{pp}}}}{{{\rm{USA}}}}})}^{{{{\rm{elasticity}}}}}$$

where GNIpp = Gross national income per capita, expressed in 2023 international dollars (purchasing power parity); elasticity = income elasticity of VSL

We estimate VSLY_Malawi_ as **$425.96** and **$2,427.31** in 2023 USD terms, assuming an income elasticity of VSL of 1.5^50^ and 1^49^, respectively. These provide lower and upper bound VSLY values to test the robustness of our ROI results. Evaluating ROI across this range of VSLY values was important, as lower VSLY assumptions tend to favour lower-cost interventions, even when their overall health impact is modest, while higher VSLY values, conversely, give greater weight to strategies with larger health benefits, even if their costs are higher. Supplementary Table 3 in the appendix provides details on the estimation methodology.

### Reporting summary

Further information on research design is available in the Nature Portfolio Reporting Summary linked to this article.

## Supplementary information

Supplementary Information

Reporting Summary

Transparent Peer Review file

## Data Availability

The data used for running these analyses, including model parameters, anonymised aggregated and publicly available data, are available within a publicly available GitHub repository [https://github.com/UCL/TLOmodel]. Detailed model documentation, including parameter values, appointment footprints, and calibration methods, is available at ref. ^25^.

## Code availability

Code used for this analysis is open-source and available from GitHub at [https://github.com/UCL/TLOmodel]. The version corresponding to this manuscript is archived as release hss_htm_analysis and can be accessed at https://github.com/UCL/TLOmodel/releases/tag/hss_htm_analysis and via https://doi.org/10.5281/zenodo.20608522.
