# Supplementary material for: System-wide investments generate health and enhance HIV, TB and malaria control in Malawi
Source: Nat Commun. 2026 Sep 4;17:10541. doi: 10.1038/s41467-026-77306-5 (PMC13642251; doi:10.1038/s41467-026-77306-5)
Supplement: Supplementary file 1 [file 41467_2026_77306_MOESM1_ESM.pdf]

System-Wide Investments Generate Health And Enhance HIV, TB and Malaria Control in Malawi

Appendix

A. Additional data

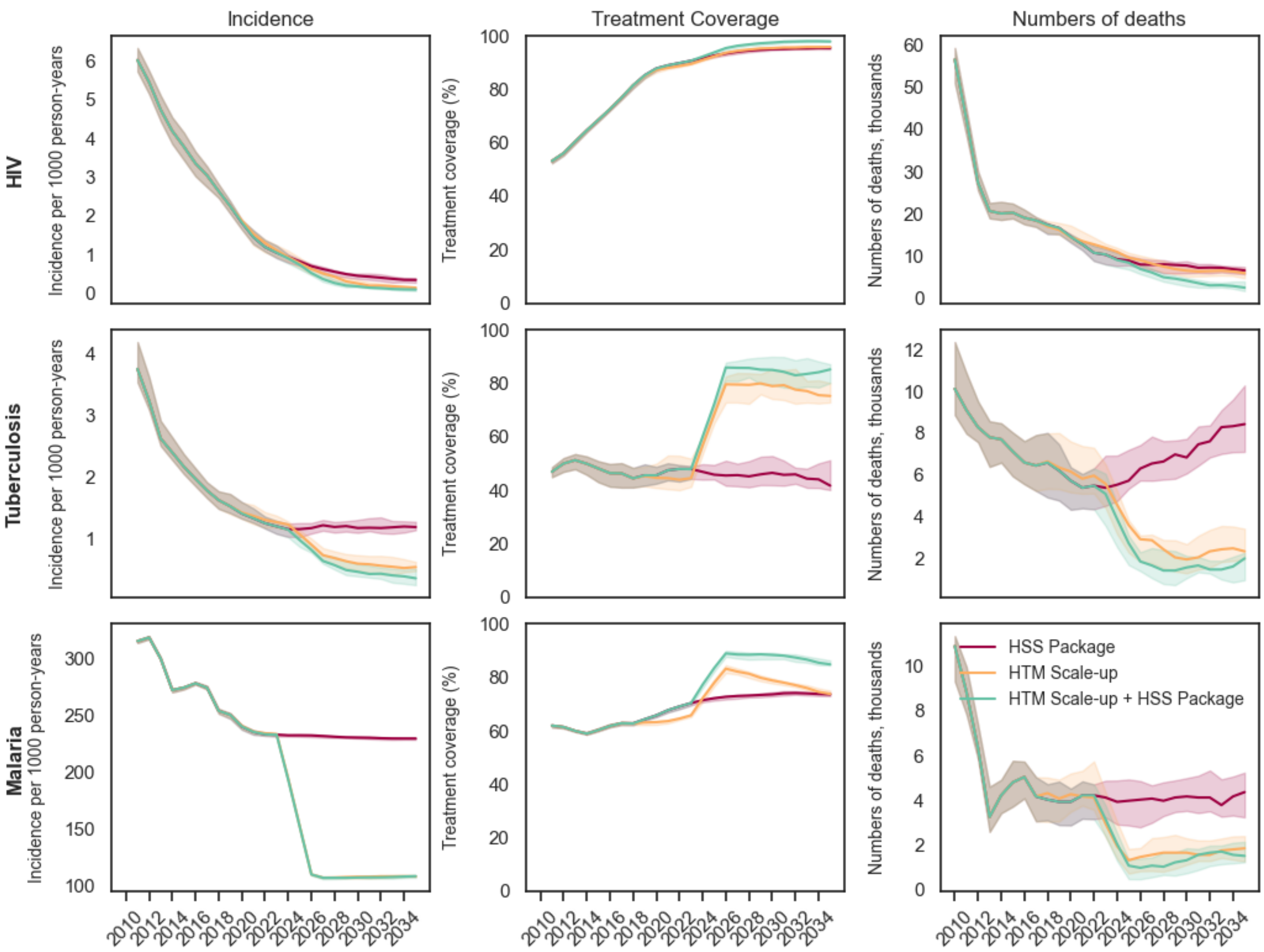

Supplementary Figure 1: The incidence per 1000 person-years (first column), the treatment coverage (second column) and the numbers of deaths (third column) for each disease (HIV first row, tuberculosis second row and malaria third row) for three scenarios: health system strengthening (HSS) Expansion Package, HIV, tuberculosis and malaria (HTM) Programs Scale-up without HSS Expansion Package, and HTM Programs Scale-up with HSS Expansion Package. Outputs are shown for each year of the simulation (2010 - 2035) with solid lines depicting the medians across 5 runs and shaded areas showing the uncertainty intervals (2.5th and 97.5th percentiles of the aggregated runs).

(a)

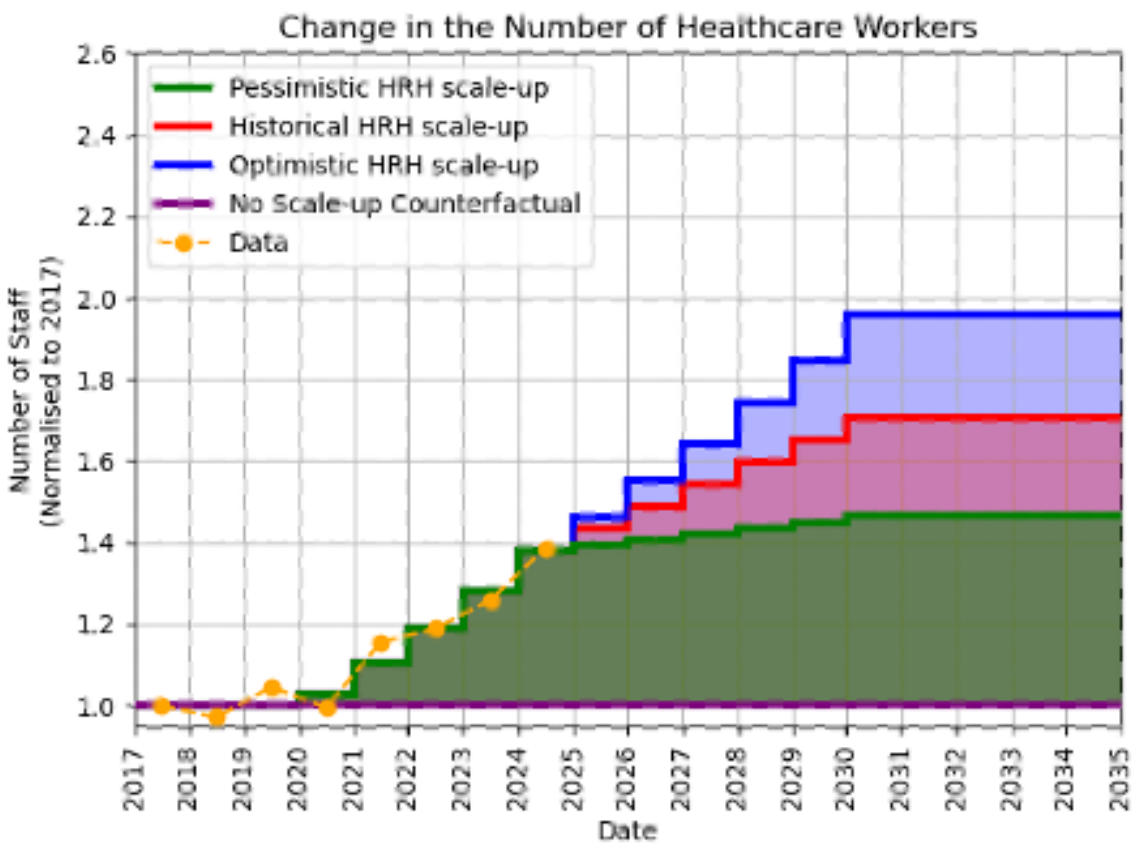

(b)

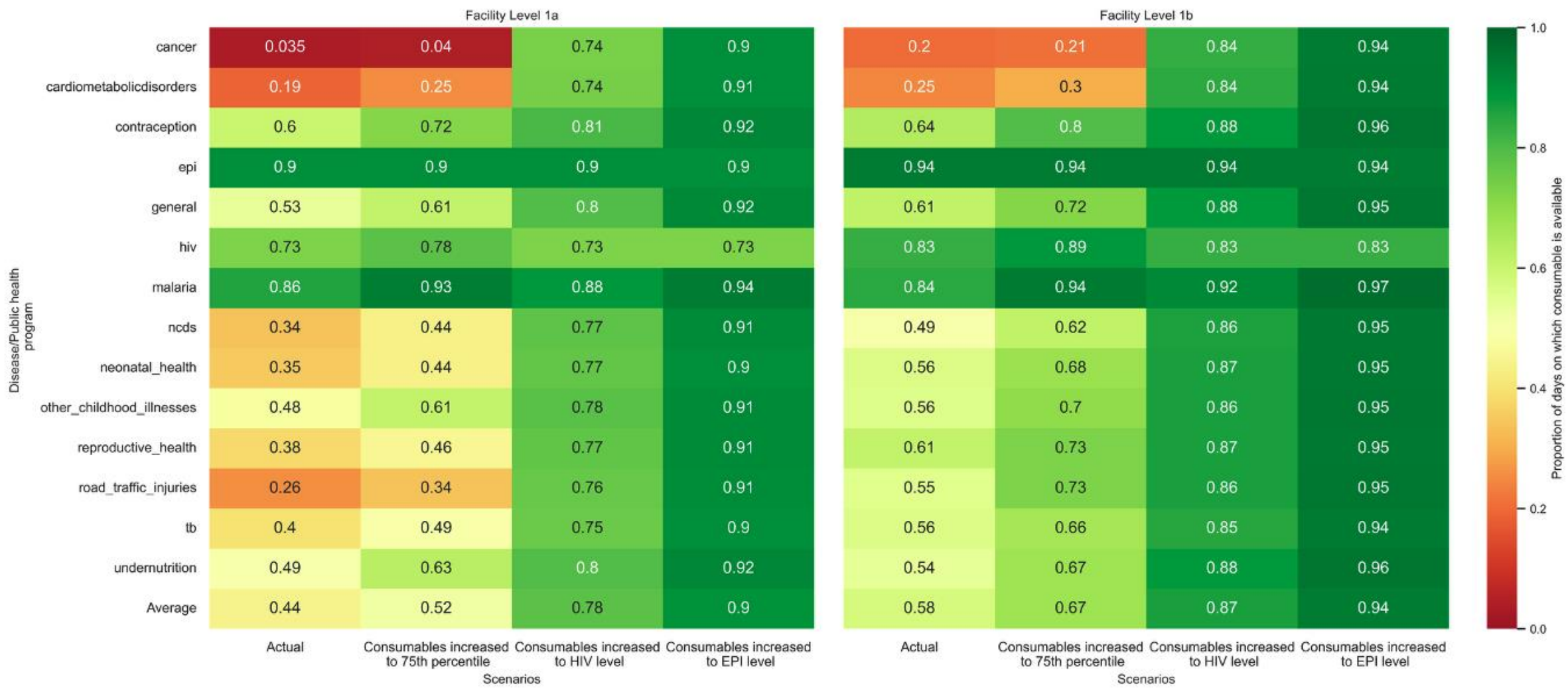

(c)

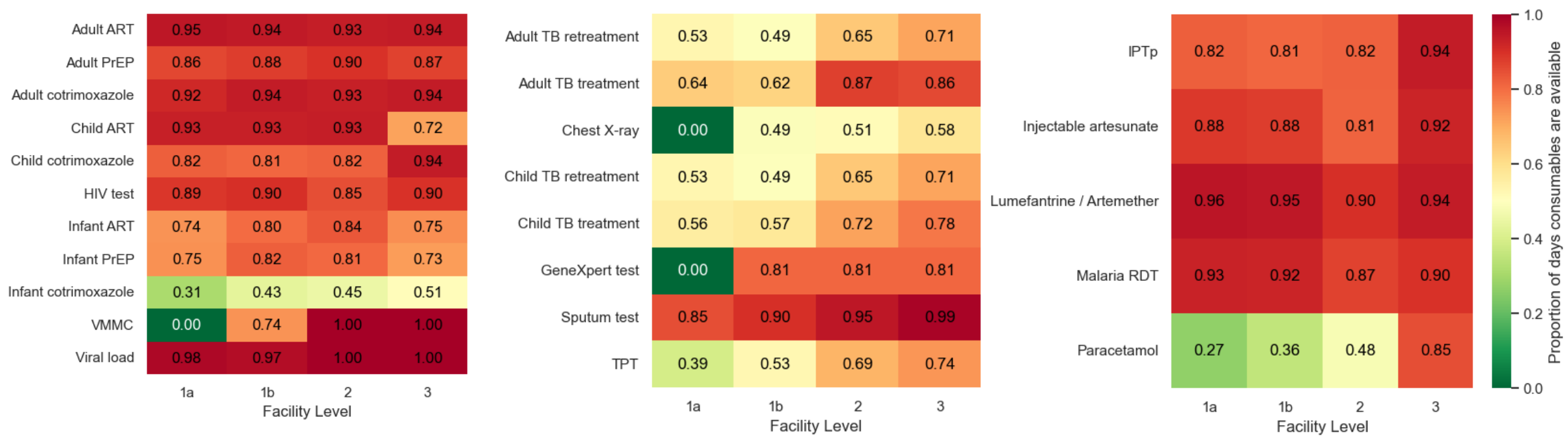

Supplementary Figure 2: The proportional increase in the number of health workers compared to 2017 levels under 4 scenarios (a); the average availability of consumables for facility levels 1a and 1b categorised by disease/public health program under 4 scenarios – Actual (used in the Baseline scenario), Increase all consumables to 75th percentile, Consumables available at HIV levels, Consumables available at EPI levels (b); the average availability of specific consumables related to HIV, TB and malaria care in facility levels 1a, 1b, 2 and 3 (c).

B. Economic analysis

1. Expansion path

The expansion path or cost-effectiveness frontier represents the set of non-dominated investment strategies, those that deliver the greatest health gains (measured in DALYs averted) for each level of additional spending relative to the baseline. Each point on the frontier reflects a cumulative investment package, building incrementally from lower-cost to higher-cost options. Strategies lying to the left or above the frontier are considered inefficient, as greater health impact could be achieved for the same or lower cost by adopting one of the frontier strategies.

The frontier allows decision-makers to identify the most efficient use of resources under varying budget constraints, and to evaluate the marginal cost-effectiveness of moving from one investment level to the next. This approach aligns with standard economic evaluation frameworks on how to prioritise interventions under constrained health budgets[1,2]. Instead of comparing specific combinations of investment options (as in Figure 2 in the manuscript), here we consider an exhaustive list of combinations of five options – HIV programme scale-up, TB programme scale-up, Malaria programme scale-up, Consumables increased to the 75th percentile, Historical HRH Scale-up. By identifying the optimum expansion path (among the alternatives considered), the frontier offers practical guidance for policymakers on which options to prioritise in what sequence. The resulting path includes: (1) Standalone TB program scale-up (2) TB program scale-up with consumables increased to the 75th percentile; (3) TB program scale-up with the full HSS Expansion Package; and (4) joint TB and malaria program scale-up with the full HSS Expansion Package.

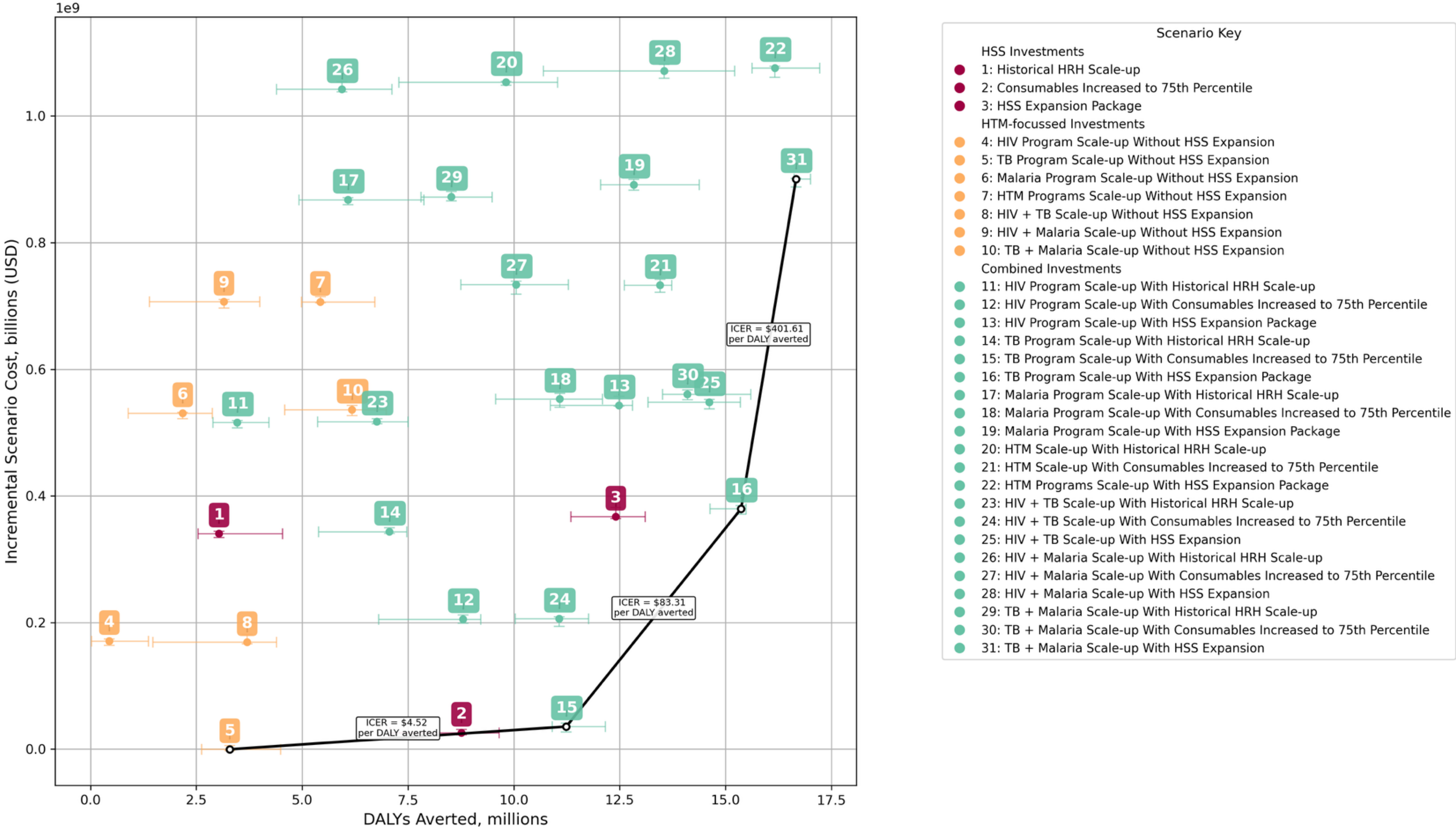

Supplementary Figure 3: Expansion path of incremental investments in disease program expansion and health system strengthening options. Values shown are the median of five runs with uncertainty intervals derived from the 2.5th and 97.5th percentiles. *Note: The incremental scenario costs and DALYs averted shown on the plot are relative to the baseline scenario. The ICER labels on the expansion path represent incremental cost-effectiveness ratios between successive scenarios on the frontier, rather than relative to the baseline.*

2. **Incremental costs**

We estimated incremental service level costs (SC) for each investment scenario across five cost categories: human resources for health, medical consumables, medical equipment, facility operations, and malaria scale-up. The costing methodology for the first four categories is detailed in Mohan et al. (2025)[3], based on the latest available local cost data for Malawi. The costs associated with malaria scale-up - including insecticide-treated bednet distribution and indoor residual spraying (IRS) - were estimated using figures from Stelmach et al. (2018)[4] , updated with recent consumable price data from Malawi. Specifically, we assumed a cost of $1.40 per person covered by a bednet per year (based on a 3-year lifespan and an average of 1.8 people per bednet) and $3.50 per person covered by IRS annually. Supplementary Table 1 presents a breakdown of the median incremental SC across these categories for each scenario, relative to the Baseline.

Supplementary Table 1: Median incremental service-level cost by scenario (2025-2035)

Scenario	Human resources for health	Medical consumables	Medical equipment	Facility operations	Malaria scale-up	Total
Pessimistic HRH Scale-up	102,171,081.74	17,091,236.19	-	-	-	119,262,317.93
Historical HRH Scale-up	292,081,826.14	50,439,484.06	-	-	-	342,521,310.20
Optimistic HRH Scale-up	682,094,725.64	98,688,909.45	-	-	-	780,783,635.09
Consumables Increased to 75th Percentile	-	29,092,851.21	-	(82.27)	-	29,092,768.94
Consumables Increased to HIV levels	-	95,889,605.72	-	60,875.86	-	95,950,481.59
Consumables Increased to EPI levels	-	116,026,089.68	-	60,875.86	-	116,086,965.54
HSS Expansion Package	292,081,826.14	81,338,207.67	-	-	-	373,420,033.81
HIV Program Scale-up Without HSS Expansion	-	171,589,191.72	-	-	-	171,589,191.72

HIV Program Scale-up With HSS Expansion Package	292,081,826.14	256,686,630.41	-	(82.27)	-	548,768,374.28
TB Program Scale-up Without HSS Expansion	-	3,847,186.94	-	-	-	3,847,186.94
TB Program Scale-up With HSS Expansion Package	292,081,826.14	89,111,952.50	-	(82.27)	-	381,193,696.37
Malaria Program Scale-up Without HSS Expansion	-	(7,659,666.06)	538,814,049.38	(82.27)	-	531,154,301.05
Malaria Program Scale-up With HSS Expansion Package	292,081,826.14	72,186,087.53	531,649,150.59	(82.27)	-	895,916,981.98
HTM Programs Scale-up Without HSS Expansion	-	167,551,835.55	542,119,451.21	-	-	709,671,286.76
HTM Programs Scale-up With HSS Expansion Package	292,081,826.14	250,778,967.21	534,472,554.56	(82.27)	-	1,077,333,265.63

3. Projected health spending

Projected total health spending under the Baseline scenario was estimated to contextualize the scale of incremental above service level costs.
To obtain these, per capita health spending projections from Dieleman et al. (2018)[5] were multiplied with annual population projections generated by the Thanzi La Onse (TLO) model (under the baseline scenario) for the years 2025–2035. The per capita health spending projections include health spending from all sources - government, development assistance, prepaid private spending (insurance), and out of pocket spending. Supplementary table 2 presents estimates of projected health spending for each year between 2025 and 2035 along with the discounted cumulative health spending over the full projection period, applying a 3% annual discount rate to align with our economic evaluation estimates.

Supplementary Table 2: Projections of health spending (Baseline scenario)

Year	Health spending per capita (2023 USD)	Projected population (median value)	Projected total health spending
	(A)	(B)	(A) X (B)
2025	66.58	22,708,106.73	1,511,914,511.11
2026	67.25	23,430,725.30	1,575,627,001.59
2027	67.92	24,173,844.72	1,641,854,940.45
2028	68.60	24,911,439.08	1,708,870,868.84
2029	69.28	25,679,130.44	1,779,148,164.56
2030	69.98	26,494,075.52	1,853,966,777.16
2031	70.68	27,302,186.99	1,929,620,871.61
2032	71.38	28,152,754.11	2,009,633,271.26
2033	72.10	28,988,781.63	2,090,004,694.60
2034	72.82	29,874,680.01	2,175,414,078.40
2035	73.55	30,784,568.74	2,264,087,048.32
Grand total projected health spending (discounted at 3%)			20,540,142,227.92

3. Value of a Statistical Life Year (VSLY)

Our Return on Investment (ROI) calculations rely upon estimates of the Value of a Statistical Life Year (VSLY) to monetise the health impact of scenarios, measured in terms of Disability-adjusted Life years (DALYs) lost averted. Our main results use the VSLY figure of $834 used by the Government of Malawi[6]. Given the inherent uncertainty in assigning a monetary value to health benefits, alternative VSLY estimates were considered. These were estimated using the methodology outlined by Robinson et al. (2019)[7]. Supplementary Table 3 describes the formulae used to estimate the alternative VSLY estimates used in our sensitivity analysis. Our estimates apply two alternative assumptions for the income elasticity of the Value of a Statistical Life (VSL) - 1.0 and 1.5 - reflecting guidance from previous literature[7,8]. These estimates provide us an upper and lower limit for VSLY around value of $834 used for our main results. Gross national income per capita (GNIpp) figures were sourced from World Development Indicators (2023) for Malawi and the United States, with adjustments made for purchasing power parity and currency conversions to 2023 USD. Conditional life expectancy at age 30–34 in 2025 was used to convert VSL estimates into VSLY estimates. Life expectancy was estimated using the TLO model *Baseline* scenario, extracting the age-specific all-cause mortality rates for that year and using standard life tables to estimate the life expectancy conditional on survival by age[9].

Evaluating ROI across this range of VSLY values was important, as lower VSLY assumptions tend to favour lower-cost interventions, even when their overall health impact is modest, while higher VSLY values, conversely, give greater weight to strategies with larger health benefits, even if their costs are higher. Supplementary Table 3 provides details on the estimation methodology.

Supplementary Table 3: Estimation of Value of Statistical Life Year (VSLY) For Malawi

Indicator	Value	Unit	Source
$GNIpp_{USA}$	57,800	2017 PPP Dollars	Robinson et al (2020)
VSL_{USA}	9,400,000	2017 PPP Dollars	Robinson et al (2020)
$GNIpp_{Malawi}$	1,780	2023 PPP Dollars	World Development Indicators (2023)
LE_0	62.8	years	TLO Model projection (Baseline scenario)
$LE_{30\text{-}34}$	40.2	years	TLO Model projection (Baseline scenario)
$VSL_{Malawi\|elasticity = 1}$	289,481	2023 US Dollars	$(VSL_{USA} * (\frac{GNIpp_{Malawi}}{GNIpp_{USA}})^{elasticity}) * conversion\ factor$
$VSL_{Malawi\|elasticity = 1.5}$	50,800.28	2023 US Dollars	$(VSL_{USA} * (\frac{GNIpp_{Malawi}}{GNIpp_{USA}})^{elasticity}) * conversion\ factor$
$VSLY_{Malawi\|elasticity = 1}$	2427.31	2023 US Dollars	$\frac{VSL_{Malawi\|elastcity = 1}}{LE_{30-34}}$

$VSLY_{Malawi\|elasticity\ =\ 1.5}$	425.96	2023 US Dollars	$\frac{VSL_{Malawi\|elastcity = 1.5}}{LE_{30-34}}$

Key: GNIpp = Gross national income per capita; PPP = Purchasing power parity; VSL = Value of Statistical Life; VSLY = Value of Statistical Life Year; LE_0 *= Life expectancy at birth;* $LE_{30\text{-}34}$ *= Conditional life expectancy at 30-34 years of age; conversion factor = 2023 PPP dollars to 2023 US Dollars conversion rate (assumed to be 0.3371); elasticity = income elasticity of VSL*
Sources: Robinson et al (2019)[7]*, World Development Indicators (2023)*[10]

C. Sensitivity analysis

1. Alternative discount rates

Health and economic outcomes were evaluated under multiple discounting assumptions to account for different time preferences regarding the value of costs and benefits. This can affect the valuation of scenarios whose health impact and cost are not evenly distributed over time. For example, a high discount rate would reduce the value of scenarios for which larger health gains are made during future years. Consistent with standard economic evaluations, the primary analysis applied a 3% annual discount rate to both health and costs[11]. Following WHO CHOICE methods, results were additionally reported with zero discounting of health outcome and 3% discounting of costs[2]. Finally, recognising the potential for higher growth rate in low-income countries, we reported results assuming a 5% discount rate for both health and costs[11]. Estimating ICER and ROI under varying discount rate assumptions enabled us to assess the robustness of our findings to different societal time preferences regarding the value of future costs and health benefits.

Supplementary Figures 4 and 5 present the cost-effectiveness plane plots of incremental costs versus DALYs averted across all scenarios under alternative discount rate assumptions. Supplementary Figure 4 applies a 0% discount rate for health outcomes and a 3% discount rate for cost, while Supplementary Figure 5 applies a 5% discount rate for both health outcomes and costs. These alternative discounting assumptions allow us to test the sensitivity of our findings to different valuations of future health benefits and costs. Across both alternative discounting scenarios, the relative cost-effectiveness ranking of the different investment strategies remains qualitatively unchanged from our main results, supporting the robustness of our main conclusions.

Supplementary Figure 6 and Supplementary Tables 4 and 5 present ROI estimates for the joint HTM scenario under the three approaches assuming alternative discount rates and a VSLY of $834.

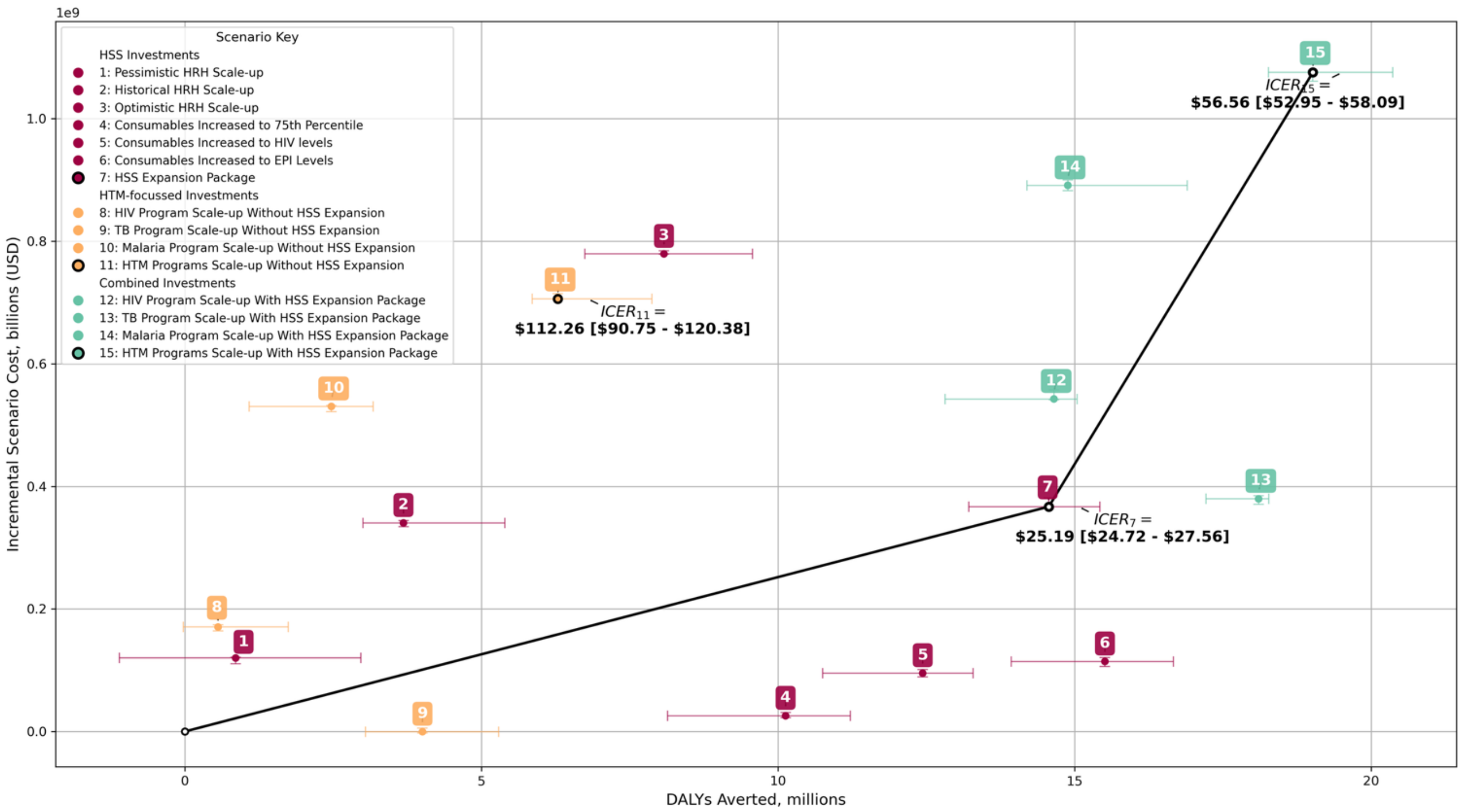

Supplementary Figure 4: DALYs averted and incremental costs (compared to baseline) over the 11-year period (2025-2035). Values shown are the median of five runs with uncertainty intervals derived from the 2.5th and 97.5th percentiles. *Note: these findings assume 0% discount rate for health and 3% discount rate for costs.*

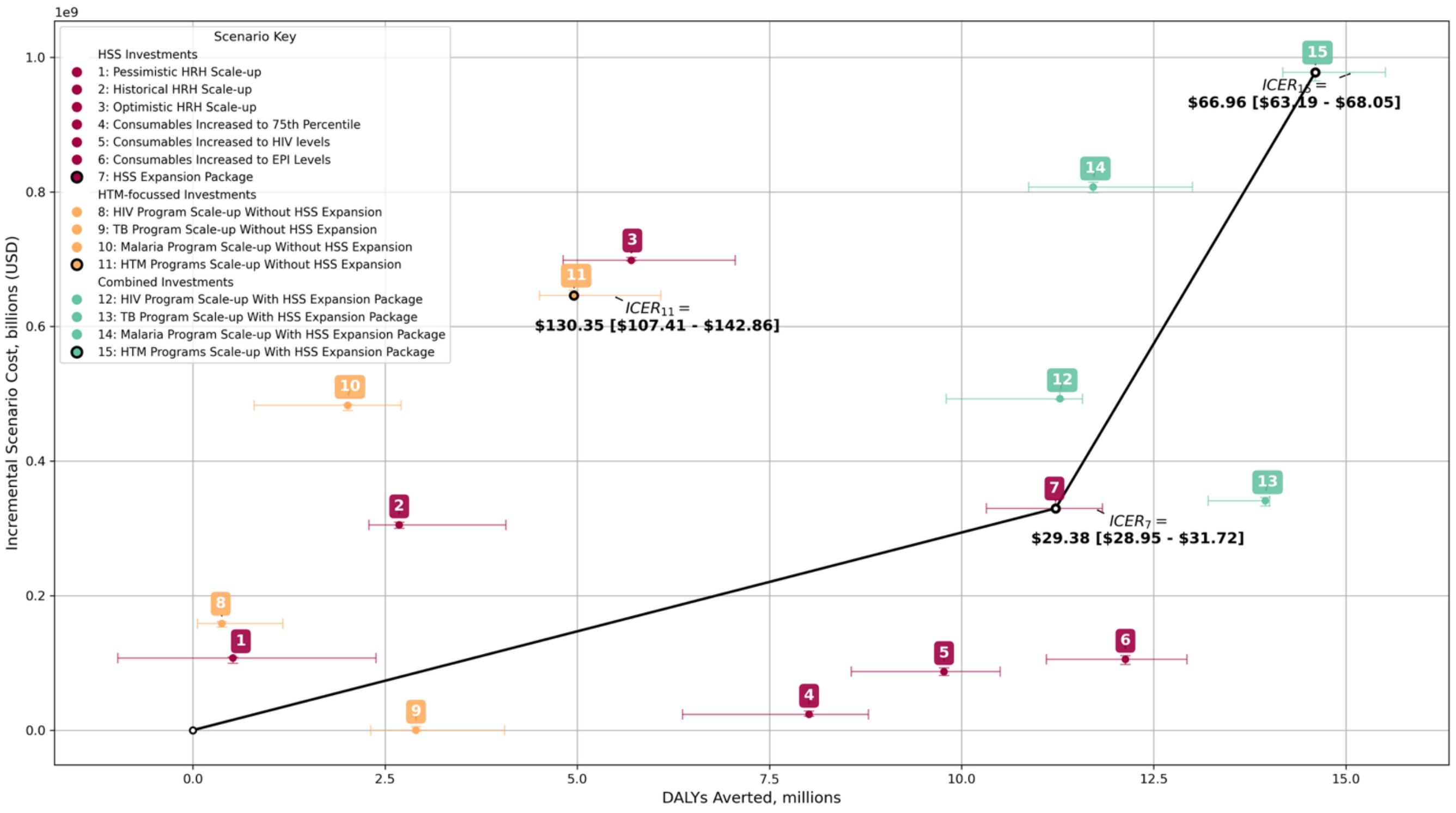

Supplementary Figure 5: DALYs averted and incremental costs (compared to baseline) over the 11-year period (2025-2035). Values shown are the median of five runs with uncertainty intervals derived from the 2.5th and 97.5th percentiles. *Note: these findings assume 5% discount rate for both health and costs.*

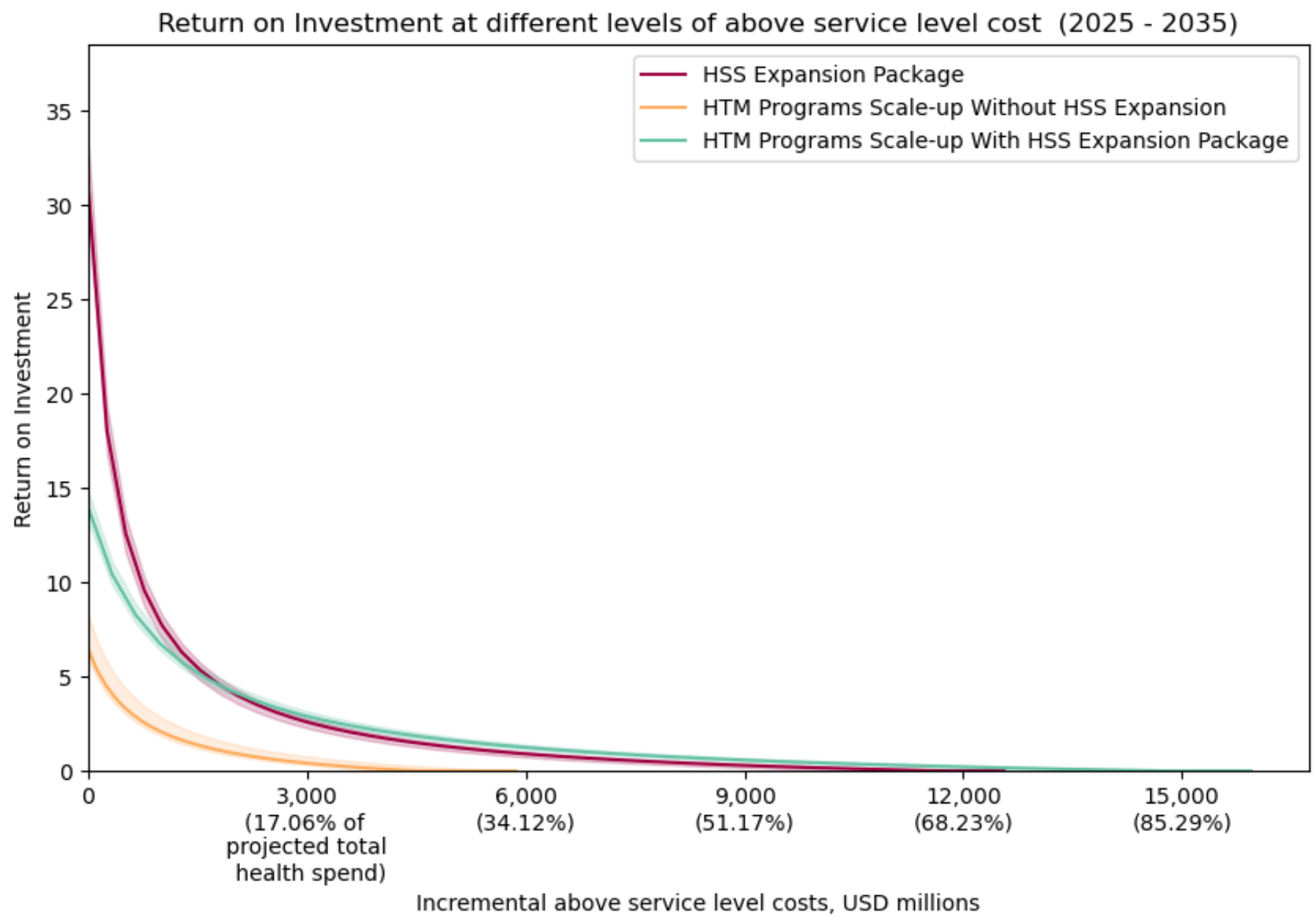

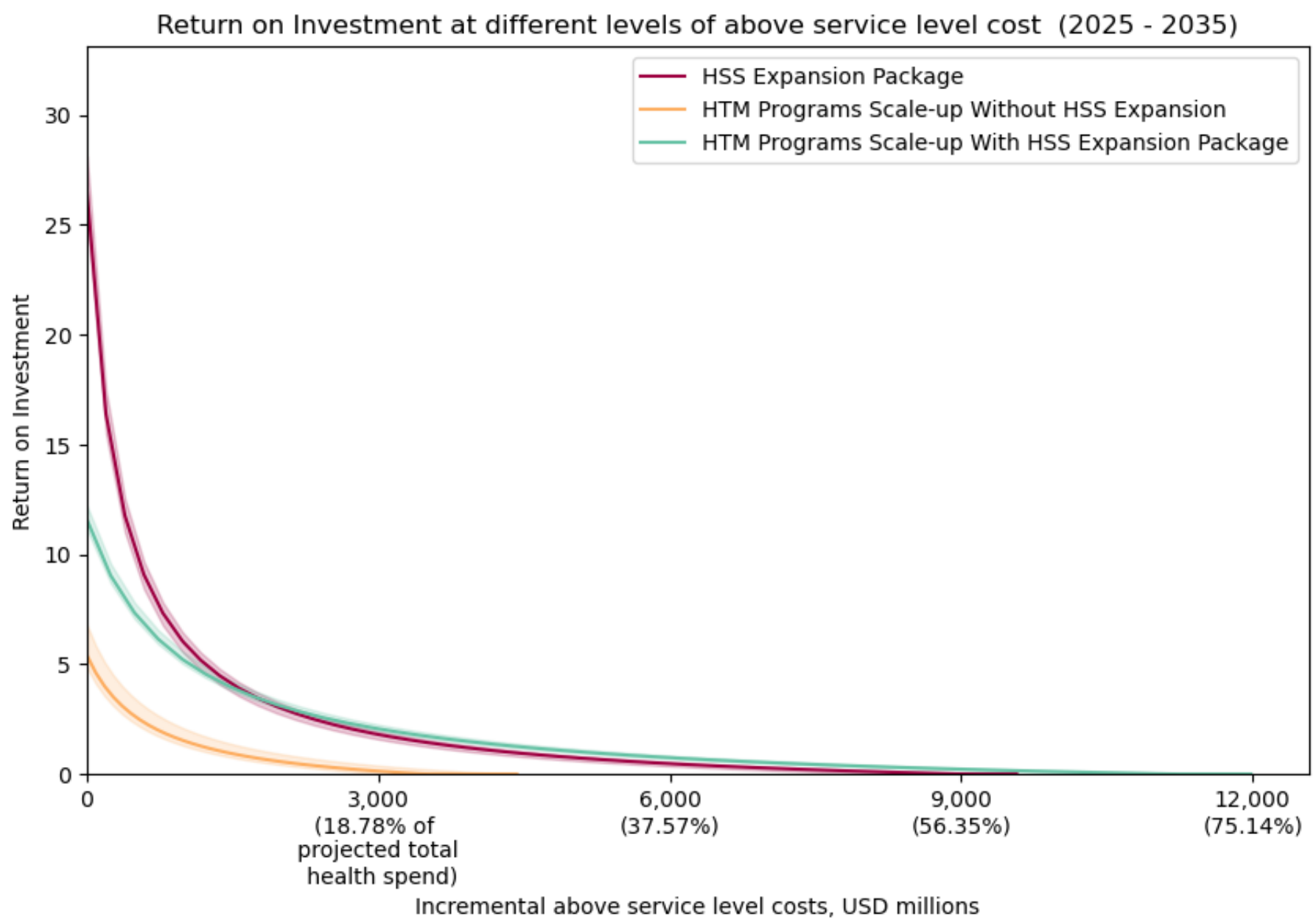

Supplementary Figure 6. Return on Investment (ROI) assuming alternative discount rates. Upper panel shows ROI estimates with 0% discount rate for health and 3% discount rate for costs; lower panel shows 5% discount rate for health and 5% discount rate for costs. Values shown are the median of five runs with uncertainty intervals derived from the 2.5th and 97.5th percentiles. *Note: ROI estimates are clipped at a minimum value of 0. Value of statistical life year (VSLY) is assumed to be $834.*

Supplementary Table 4. Return on Investment by Scenario (Value of Statistical Life Year = $834; Health discount rate = 0% and cost discount rate = 3%)

Scenario	Monetised health benefits ($, billion)	Service-level costs ($, million)	Above Service-level costs ($, million) *(assumed to be equal to 138% of service-level costs)*	ROI (assuming zero above service level costs)	ROI (assuming non-zero above service level costs)
Pessimistic HRH Scale-up	$0.72 [$0.00 - $2.48]	$119.97 [$110.59 - $121.04]	$165.56 [$152.62 - $167.04]	5.04 [-1.0 - 19.46]	1.54 [-1.0 - 7.6]
Historical HRH Scale-up	$3.08 [$2.51 - $4.50]	$340.27 [$334.23 - $344.35]	$469.57 [$461.23 - $475.20]	8.06 [6.5 - 12.07]	2.81 [2.15 - 4.49]
Optimistic HRH Scale-up	$6.74 [$5.63 - $7.98]	$779.35 [$778.99 - $784.63]	$1,075.51 [$1,075.01 - $1,082.79]	7.59 [6.22 - 9.2]	2.61 [2.03 - 3.28]
Consumables Increased to 75th Percentile	$8.45 [$6.79 - $9.36]	$25.59 [$22.54 - $31.12]	$35.31 [$31.10 - $42.95]	283.23 [228.89 - 411.72]	118.42 [95.59 - 172.41]
Consumables Increased to HIV levels	$10.38 [$8.97 - $11.09]	$95.20 [$89.01 - $100.96]	$131.37 [$122.83 - $139.33]	108.19 [92.57 - 122.73]	44.88 [38.31 - 50.99]
Consumables Increased to EPI Levels	$12.94 [$11.62 - $13.90]	$114.35 [$106.27 - $120.84]	$157.80 [$146.65 - $166.76]	113.34 [100.72 - 122.99]	47.04 [41.74 - 51.1]
**HSS Expansion Package**	**$12.15 [$11.02 - $12.87]**	**$366.95 [$364.19 - $381.44]**	**$506.39 [$502.58 - $526.39]**	**30.82 [29.27 - 33.14]**	**12.37 [11.72 - 13.34]**
HIV Program Scale-up Without HSS Expansion	$0.47 [$0.03 - $1.46]	$170.46 [$164.08 - $173.96]	$235.24 [$226.44 - $240.07]	1.69 [-0.82 - 7.45]	0.13 [-0.92 - 2.55]
TB Program Scale-up Without HSS Expansion	$3.34 [$2.54 - $4.42]	$-0.33 [$-2.22 - $5.78]	$-0.46 [$-3.06 - $7.97]	1093.17 [766.33 - 10923.88]	459.9 [321.41 - 4590.44]
Malaria Program Scale-up Without HSS Expansion	$2.06 [$0.91 - $2.65]	$530.42 [$521.81 - $532.04]	$731.98 [$720.10 - $734.22]	2.89 [0.73 - 3.98]	0.63 [-0.27 - 1.09]

**HTM Programs Scale-up Without HSS Expansion**	**$5.25 [$4.88 - $6.57]**	**$706.15 [$705.04 - $714.71]**	**$974.49 [$972.96 - $986.30]**	**6.43 [5.92 - 8.21]**	**2.12 [1.91 - 2.87]**
HIV Program Scale-up With HSS Expansion Package	$12.22 [$10.69 - $12.55]	$542.65 [$541.61 - $557.39]	$748.85 [$747.42 - $769.19]	21.16 [18.71 - 21.55]	8.31 [7.28 - 8.47]
TB Program Scale-up With HSS Expansion Package	$15.10 [$14.36 - $15.24]	$379.67 [$371.05 - $384.69]	$523.95 [$512.05 - $530.88]	38.48 [36.83 - 39.79]	15.59 [14.89 - 16.14]
Malaria Program Scale-up With HSS Expansion Package	$12.42 [$11.84 - $14.10]	$891.22 [$882.74 - $899.92]	$1,229.89 [$1,218.19 - $1,241.89]	12.93 [12.32 - 14.95]	4.85 [4.6 - 5.7]
**HTM Programs Scale-up With HSS Expansion Package**	**$15.86 [$15.24 - $16.99]**	**$1,075.65 [$1,061.28 - $1,078.40]**	**$1,484.39 [$1,464.56 - $1,488.20]**	**13.95 [13.19 - 14.75]**	**5.28 [4.96 - 5.62]**

Supplementary Table 5. Return on Investment by Scenario (Value of Statistical Life Year = $834; Health and cost discount rate = 5%)

Scenario	Monetised health benefits ($, billion)	Service-level costs ($, million)	Above Service-level costs ($, million) *(assumed to be equal to 138% of service-level costs)*	ROI (assuming zero above service level costs)	ROI (assuming non-zero above service level costs)
Pessimistic HRH Scale-up	$0.44 [$0.00 - $1.99]	$107.27 [$99.47 - $108.65]	$148.04 [$137.26 - $149.94]	3.1 [-1.0 - 17.3]	0.72 [-1.0 - 6.69]
Historical HRH Scale-up	$2.24 [$1.91 - $3.39]	$305.10 [$300.10 - $308.98]	$421.03 [$414.14 - $426.39]	6.36 [5.36 - 9.99]	2.09 [1.67 - 3.62]
Optimistic HRH Scale-up	$4.76 [$4.02 - $5.88]	$698.33 [$697.50 - $702.79]	$963.69 [$962.55 - $969.85]	5.82 [4.75 - 7.39]	1.87 [1.42 - 2.53]
Consumables Increased to 75th Percentile	$6.69 [$5.31 - $7.33]	$23.52 [$20.59 - $28.63]	$32.46 [$28.41 - $39.51]	247.71 [188.97 - 353.11]	103.5 [78.82 - 147.78]
Consumables Increased to HIV levels	$8.15 [$7.15 - $8.76]	$86.92 [$81.42 - $92.61]	$119.95 [$112.35 - $127.80]	93.06 [79.98 - 105.84]	38.52 [33.02 - 43.89]
Consumables Increased to EPI Levels	$10.12 [$9.26 - $10.78]	$105.29 [$97.47 - $110.75]	$145.30 [$134.50 - $152.83]	97.7 [86.97 - 104.06]	40.47 [35.96 - 43.14]
**HSS Expansion Package**	**$9.36 [$8.61 - $9.87]**	**$329.70 [$327.45 - $342.56]**	**$454.99 [$451.88 - $472.74]**	**26.5 [25.27 - 28.14]**	**10.55 [10.04 - 11.25]**
HIV Program Scale-up Without HSS Expansion	$0.31 [$0.05 - $0.98]	$158.37 [$152.93 - $161.45]	$218.55 [$211.05 - $222.80]	0.95 [-0.68 - 5.08]	-0.18 [-0.86 - 1.56]
TB Program Scale-up Without HSS Expansion	$2.42 [$1.93 - $3.38]	$-0.12 [$-1.42 - $5.40]	$-0.16 [$-1.96 - $7.45]	1389.5 [629.09 - 22612.18]	584.41 [263.74 - 9501.5]
Malaria Program Scale-up Without HSS Expansion	$1.68 [$0.66 - $2.26]	$482.72 [$475.25 - $484.39]	$666.15 [$655.84 - $668.46]	2.48 [0.39 - 3.67]	0.46 [-0.42 - 0.96]
**HTM Programs Scale-up Without HSS Expansion**	**$4.13 [$3.76 - $5.08]**	**$646.12 [$644.32 - $653.70]**	**$891.65 [$889.16 - $902.10]**	**5.4 [4.83 - 6.78]**	**1.69 [1.45 - 2.27]**
HIV Program Scale-up With HSS Expansion Package	$9.41 [$8.17 - $9.65]	$492.61 [$491.36 - $506.33]	$679.81 [$678.08 - $698.74]	17.75 [15.6 - 18.15]	6.88 [5.97 - 7.04]

TB Program Scale-up With HSS Expansion Package	$11.64 [$11.02 - $11.68]	$340.93 [$333.16 - $345.81]	$470.48 [$459.76 - $477.22]	33.13 [31.21 - 33.97]	13.34 [12.53 - 13.69]
Malaria Program Scale-up With HSS Expansion Package	$9.77 [$9.07 - $10.84]	$807.28 [$799.06 - $814.66]	$1,114.05 [$1,102.70 - $1,124.23]	11.1 [10.27 - 12.55]	4.08 [3.74 - 4.7]
**HTM Programs Scale-up With HSS Expansion Package**	**$12.18 [$11.82 - $12.94]**	**$977.74 [$964.84 - $980.35]**	**$1,349.28 [$1,331.48 - $1,352.88]**	**11.58 [11.12 - 12.2]**	**4.29 [4.09 - 4.55]**

2. Alternative Value of Statistical Life Year (VSLY)

Supplementary Figure 7 and Supplementary Tables 6 and 7 present ROI estimates for the joint HTM scenario under the three approaches assuming alternative Value of Statistical Life Year (VSLY) of $425.96 and $2427.31, applying a 3% discount rate for both health and cost.

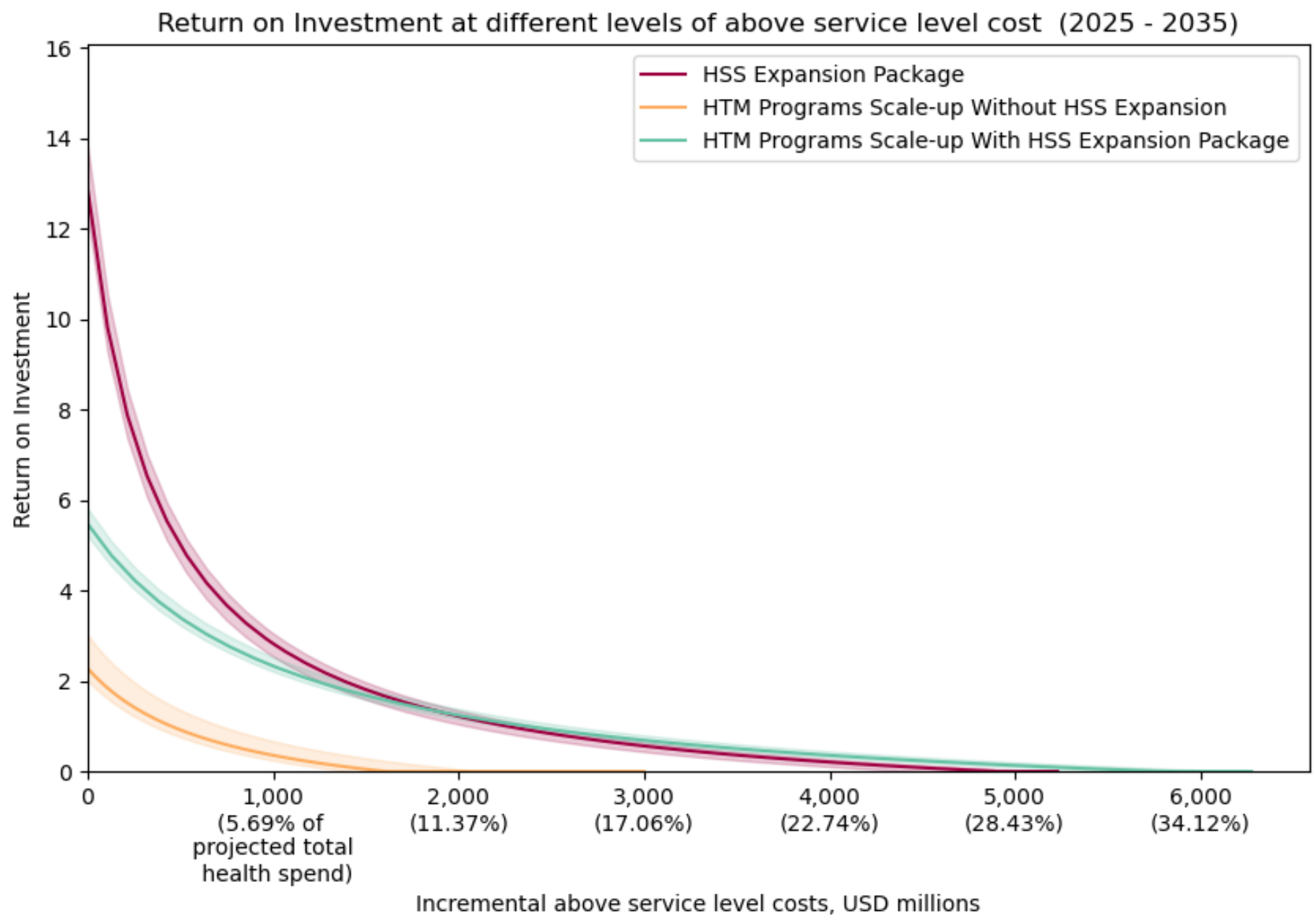

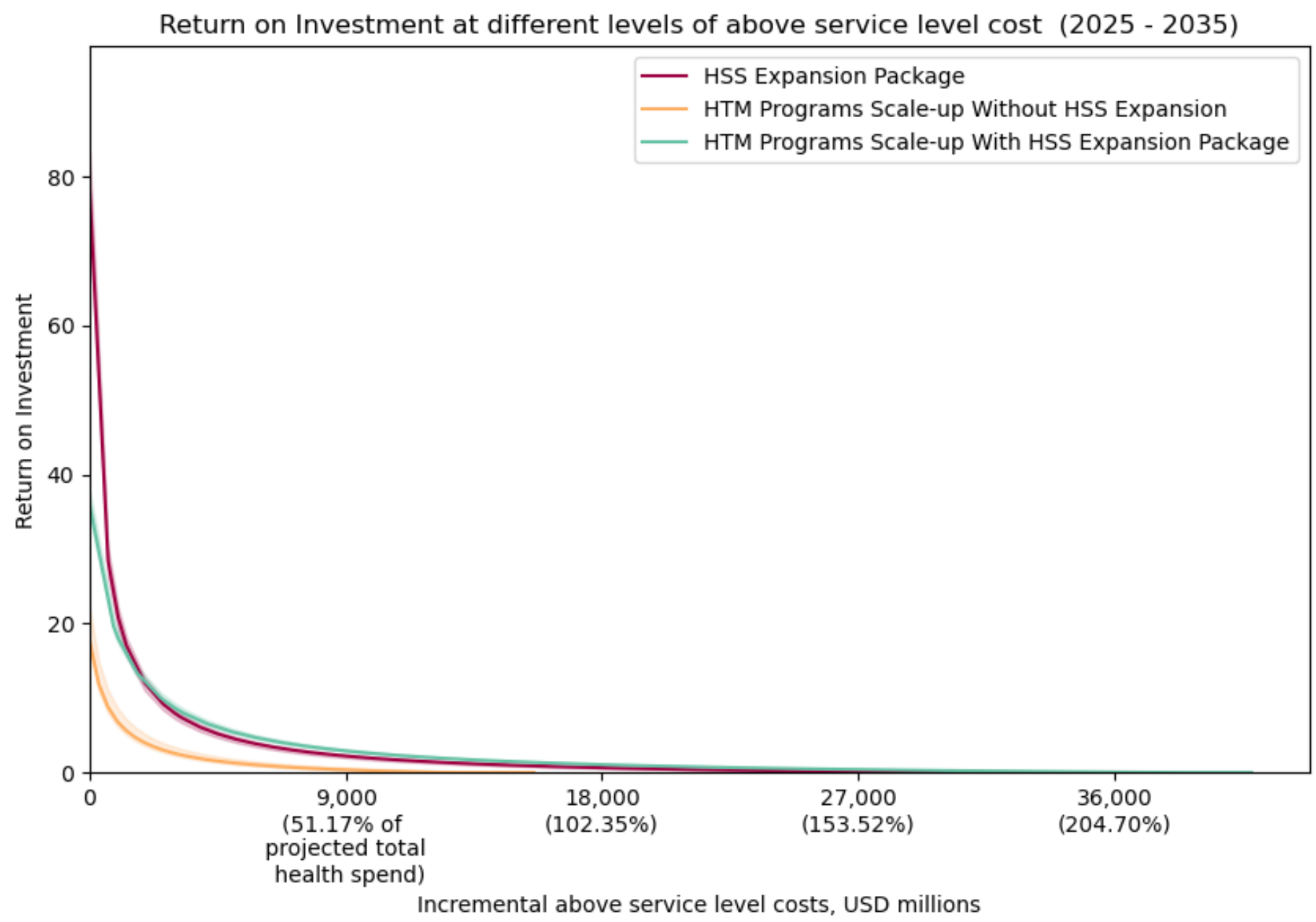

Supplementary Figure 7. Return on Investment (ROI) assuming alternative estimates of Value of Statistical Life Year (VSLY). Upper panel assumes VSLY= $425.96. Lower panel assumes VSLY= $2,427.31. Values shown are the median of five runs with uncertainty intervals derived from the 2.5th and 97.5th percentiles. * *Note: ROI estimates are clipped at a minimum value of 0. A 3% discount rate is applied to both health and costs.*

Supplementary Table 6. Return on Investment by Scenario (Value of Statistical Life Year = $425.96; Health and cost discount rate = 3%)

Scenario	Monetised health benefits ($, billion)	Service-level costs ($, million)	Above Service-level costs ($, million) *(assumed to be equal to 138% of service-level costs)*	ROI (assuming zero above service level costs)	ROI (assuming non-zero above service level costs)
Pessimistic HRH Scale-up	$0.27 [$0.00 - $1.10]	$119.97 [$110.59 - $121.04]	$165.56 [$152.62 - $167.04]	1.31 [-1.0 - 8.12]	-0.03 [-1.0 - 2.83]
Historical HRH Scale-up	$1.29 [$1.08 - $1.93]	$340.27 [$334.23 - $344.35]	$469.57 [$461.23 - $475.20]	2.81 [2.24 - 4.61]	0.6 [0.36 - 1.36]
Optimistic HRH Scale-up	$2.77 [$2.34 - $3.38]	$779.35 [$778.99 - $784.63]	$1,075.51 [$1,075.01 - $1,082.79]	2.55 [2.0 - 3.32]	0.49 [0.26 - 0.82]
Consumables Increased to 75th Percentile	$3.73 [$3.01 - $4.11]	$25.59 [$22.54 - $31.12]	$35.31 [$31.10 - $42.95]	126.54 [98.18 - 180.3]	52.59 [40.67 - 75.18]
Consumables Increased to HIV levels	$4.57 [$3.98 - $4.89]	$95.20 [$89.01 - $100.96]	$131.37 [$122.83 - $139.33]	47.19 [40.53 - 53.61]	19.25 [16.45 - 21.95]
Consumables Increased to EPI Levels	$5.68 [$5.16 - $6.07]	$114.35 [$106.27 - $120.84]	$157.80 [$146.65 - $166.76]	49.55 [44.12 - 53.16]	20.24 [17.96 - 21.76]
**HSS Expansion Package**	**$5.29 [$4.83 - $5.58]**	**$366.95 [$364.19 - $381.44]**	**$506.39 [$502.58 - $526.39]**	**12.86 [12.27 - 13.81]**	**4.82 [4.57 - 5.22]**
HIV Program Scale-up Without HSS Expansion	$0.19 [$0.01 - $0.58]	$170.46 [$164.08 - $173.96]	$235.24 [$226.44 - $240.07]	0.09 [-0.92 - 2.4]	-0.54 [-0.96 - 0.43]
TB Program Scale-up Without HSS Expansion	$1.40 [$1.12 - $1.91]	$-0.33 [$-2.22 - $5.78]	$-0.46 [$-3.06 - $7.97]	487.34 [330.83 - 4612.31]	205.34 [138.42 - 1938.52]
Malaria Program Scale-up Without HSS Expansion	$0.93 [$0.38 - $1.23]	$530.42 [$521.81 - $532.04]	$731.98 [$720.10 - $734.22]	0.75 [-0.27 - 1.31]	-0.26 [-0.69 - -0.03]
**HTM Programs Scale-up Without HSS Expansion**	**$2.31 [$2.12 - $2.86]**	**$706.15 [$705.04 - $714.71]**	**$974.49 [$972.96 - $986.30]**	**2.28 [2.01 - 3.02]**	**0.38 [0.26 - 0.69]**

HIV Program Scale-up With HSS Expansion Package	$5.32 [$4.63 - $5.45]	$542.65 [$541.61 - $557.39]	$748.85 [$747.42 - $769.19]	8.62 [7.53 - 8.82]	3.04 [2.58 - 3.12]
TB Program Scale-up With HSS Expansion Package	$6.55 [$6.23 - $6.59]	$379.67 [$371.05 - $384.69]	$523.95 [$512.05 - $530.88]	16.37 [15.39 - 16.72]	6.3 [5.89 - 6.44]
Malaria Program Scale-up With HSS Expansion Package	$5.47 [$5.13 - $6.12]	$891.22 [$882.74 - $899.92]	$1,229.89 [$1,218.19 - $1,241.89]	5.13 [4.77 - 5.93]	1.58 [1.43 - 1.91]
**HTM Programs Scale-up With HSS Expansion Package**	**$6.89 [$6.66 - $7.34]**	**$1,075.65 [$1,061.28 - $1,078.40]**	**$1,484.39 [$1,464.56 - $1,488.20]**	**5.47 [5.2 - 5.81]**	**1.72 [1.61 - 1.86]**

Table 7. Return on Investment by Scenario (Value of Statistical Life Year = $2,427.31; Health and cost discount rate = 3%)

Scenario	Monetised health benefits ($, billion)	Service-level costs ($, million)	Above Service-level costs ($, million) *(assumed to be equal to 138% of service-level costs)*	ROI (assuming zero above service level costs)	ROI (assuming non-zero above service level costs)
Pessimistic HRH Scale-up	$1.56 [$0.00 - $6.29]	$119.97 [$110.59 - $121.04]	$165.56 [$152.62 - $167.04]	12.16 [-1.0 - 51.0]	4.53 [-1.0 - 20.85]
Historical HRH Scale-up	$7.37 [$6.18 - $11.01]	$340.27 [$334.23 - $344.35]	$469.57 [$461.23 - $475.20]	20.73 [17.47 - 30.98]	8.13 [6.76 - 12.44]
Optimistic HRH Scale-up	$15.76 [$13.34 - $19.28]	$779.35 [$778.99 - $784.63]	$1,075.51 [$1,075.01 - $1,082.79]	19.23 [16.1 - 23.63]	7.5 [6.19 - 9.35]
Consumables Increased to 75th Percentile	$21.27 [$17.18 - $23.43]	$25.59 [$22.54 - $31.12]	$35.31 [$31.10 - $42.95]	725.81 [564.19 - 1032.13]	304.38 [236.48 - 433.09]
Consumables Increased to HIV levels	$26.02 [$22.69 - $27.88]	$95.20 [$89.01 - $100.96]	$131.37 [$122.83 - $139.33]	273.62 [235.68 - 310.19]	114.39 [98.45 - 129.75]
Consumables Increased to EPI Levels	$32.35 [$29.40 - $34.60]	$114.35 [$106.27 - $120.84]	$157.80 [$146.65 - $166.76]	287.06 [256.1 - 307.62]	120.03 [107.03 - 128.67]
**HSS Expansion Package**	**$30.12 [$27.54 - $31.80]**	**$366.95 [$364.19 - $381.44]**	**$506.39 [$502.58 - $526.39]**	**77.98 [74.6 - 83.38]**	**32.19 [30.76 - 34.45]**
HIV Program Scale-up Without HSS Expansion	$1.08 [$0.08 - $3.33]	$170.46 [$164.08 - $173.96]	$235.24 [$226.44 - $240.07]	5.22 [-0.52 - 18.35]	1.61 [-0.8 - 7.13]
TB Program Scale-up Without HSS Expansion	$7.99 [$6.36 - $10.91]	$-0.33 [$-2.22 - $5.78]	$-0.46 [$-3.06 - $7.97]	2772.37 [1889.91 - 26278.28]	1165.44 [793.5 - 11041.87]
Malaria Program Scale-up Without HSS Expansion	$5.29 [$2.17 - $6.99]	$530.42 [$521.81 - $532.04]	$731.98 [$720.10 - $734.22]	8.98 [3.15 - 12.15]	3.19 [0.74 - 4.52]

**HTM Programs Scale-up Without HSS Expansion**	**$13.18 [$12.11 - $16.31]**	**$706.15 [$705.04 - $714.71]**	**$974.49 [$972.96 - $986.30]**	**17.67 [16.16 - 21.88]**	**6.84 [6.21 - 8.61]**
HIV Program Scale-up With HSS Expansion Package	$30.30 [$26.37 - $31.07]	$542.65 [$541.61 - $557.39]	$748.85 [$747.42 - $769.19]	53.84 [47.61 - 54.93]	22.04 [19.42 - 22.5]
TB Program Scale-up With HSS Expansion Package	$37.30 [$35.52 - $37.58]	$379.67 [$371.05 - $384.69]	$523.95 [$512.05 - $530.88]	97.99 [92.41 - 99.95]	40.59 [38.25 - 41.42]
Malaria Program Scale-up With HSS Expansion Package	$31.16 [$29.25 - $34.90]	$891.22 [$882.74 - $899.92]	$1,229.89 [$1,218.19 - $1,241.89]	33.96 [31.9 - 38.49]	13.69 [12.82 - 15.59]
**HTM Programs Scale-up With HSS Expansion Package**	**$39.24 [$37.93 - $41.82]**	**$1,075.65 [$1,061.28 - $1,078.40]**	**$1,484.39 [$1,464.56 - $1,488.20]**	**35.89 [34.35 - 37.79]**	**14.5 [13.85 - 15.3]**

References

1. Drummond, M. F., Sculpher, M. J., Claxton, K., Stoddart, G. L. & Torrance, G. W. *Methods for the Economic Evaluation of Health Care Programmes*. (Oxford University Press (OUP), United Kingdom, 2015).
2. Bertram, M. Y., Lauer, J. A., Stenberg, K. & Edejer, T. T. T. Methods for the Economic Evaluation of Health Care Interventions for Priority Setting in the Health System: An Update From WHO CHOICE. *Int J Health Policy Manag* **10**, 673–677 (2021).
3. Mohan, S. *et al.* Method for costing a health system using a Health Systems Model. 2025.01.22.25320881 Preprint at https://doi.org/10.1101/2025.01.22.25320881 (2025).
4. Stelmach, R., Colaço, R., Lalji, S., McFarland, D. & Reithinger, R. Cost-Effectiveness of Indoor Residual Spraying of Households with Insecticide for Malaria Prevention and Control in Tanzania. *Am J Trop Med Hyg* **99**, 627–637 (2018).
5. Global Burden of Disease Health Financing Collaborator Network. Trends in future health financing and coverage: future health spending and universal health coverage in 188 countries, 2016-40. *Lancet* **391**, 1783–1798 (2018).
6. National Planning Commission. *Medium and Long-Term Impacts of a Moderate Lockdown (Social Restrictions) in Response to the COVID-19 Pandemic in Malawi: A Rapid Cost-Benefit Analysis*. https://afidep.org/publication/medium-and-long-term-impacts-of-a-moderate-lockdown-social-restrictions-in-response-to-the-covid-19-pandemic-in-malawi-a-rapid-cost-benefit-analysis/.
7. Robinson, L. A. *et al.* Reference Case Guidelines for Benefit-Cost Analysis in Global Health and Development. SSRN Scholarly Paper at https://doi.org/10.2139/ssrn.4015886 (2019).
8. Lomas, J., Claxton, K. & Ochalek, J. Accounting for country- and time-specific values in the economic evaluation of health-related projects relevant to low- and middle-income countries. *Health Policy Plan* **37**, 45–54 (2022).
9. Eayres, D. & Williams, E. S. Evaluation of methodologies for small area life expectancy estimation. *J Epidemiol Community Health* **58**, 243–249 (2004).
10. The World Bank. World Development Indicators. https://databank.worldbank.org/source/world-development-indicators.
11. Haacker, M., Hallett, T. B. & Atun, R. On discount rates for economic evaluations in global health. *Health Policy and Planning* **35**, 107–114 (2020).
