# Supplementary material for: System-wide investments generate health and enhance HIV, TB and malaria control in Malawi
Source: Nat Commun. 2026 Sep 4;17:10541. doi: 10.1038/s41467-026-77306-5 (PMC13642251; doi:10.1038/s41467-026-77306-5)
Supplement: Supplementary file 2 [file 41467_2026_77306_MOESM2_ESM.pdf]

nature portfolio

Corresponding author(s): Dr Tara Mangal

Last updated by author(s): Aug 4, 2025

Reporting Summary

Nature Portfolio wishes to improve the reproducibility of the work that we publish. This form provides structure for consistency and transparency in reporting. For further information on Nature Portfolio policies, see our Editorial Policies and the Editorial Policy Checklist.

Statistics

For all statistical analyses, confirm that the following items are present in the figure legend, table legend, main text, or Methods section.

n/a	Confirmed	
☐	☒	The exact sample size (*n*) for each experimental group/condition, given as a discrete number and unit of measurement
☒	☐	A statement on whether measurements were taken from distinct samples or whether the same sample was measured repeatedly
☒	☐	The statistical test(s) used AND whether they are one- or two-sided<br>*Only common tests should be described solely by name; describe more complex techniques in the Methods section.*
☒	☐	A description of all covariates tested
☒	☐	A description of any assumptions or corrections, such as tests of normality and adjustment for multiple comparisons
☐	☒	A full description of the statistical parameters including central tendency (e.g. means) or other basic estimates (e.g. regression coefficient) AND variation (e.g. standard deviation) or associated estimates of uncertainty (e.g. confidence intervals)
☒	☐	For null hypothesis testing, the test statistic (e.g. *F*, *t*, *r*) with confidence intervals, effect sizes, degrees of freedom and *P* value noted<br>*Give P values as exact values whenever suitable.*
☒	☐	For Bayesian analysis, information on the choice of priors and Markov chain Monte Carlo settings
☒	☐	For hierarchical and complex designs, identification of the appropriate level for tests and full reporting of outcomes
☒	☐	Estimates of effect sizes (e.g. Cohen's *d*, Pearson's *r*), indicating how they were calculated

Our web collection on statistics for biologists contains articles on many of the points above.

Software and code

Policy information about availability of computer code

Data collection	Code used for this analysis is open-source and available from GitHub at [https://github.com/UCL/TLOmodel]. The version corresponding to this manuscript is archived as release hss_htm_analysis and can be accessed at [https://github.com/UCL/TLOmodel/releases/tag/hss_htm_analysis] and via DOI: [https://doi.org/10.5281/zenodo.20608522].
Data analysis	All associated code is open-source and available at https://github.com/UCL/TLOmodel/releases.

For manuscripts utilizing custom algorithms or software that are central to the research but not yet described in published literature, software must be made available to editors and reviewers. We strongly encourage code deposition in a community repository (e.g. GitHub). See the Nature Portfolio guidelines for submitting code & software for further information.

Data

Policy information about availability of data

All manuscripts must include a data availability statement. This statement should provide the following information, where applicable:

- Accession codes, unique identifiers, or web links for publicly available datasets
- A description of any restrictions on data availability
- For clinical datasets or third party data, please ensure that the statement adheres to our policy

The data used for running these analyses, including model parameters, anonymised aggregated and publicly available data, are available within this repository [https://github.com/UCL/TLOmodel/releases/tag/hss_htm_analysis]. Detailed model documentation, including parameter values, appointment footprints, and calibration methods, is available at Hallett et al 2025.

There are no restrictions on data availability.

Research involving human participants, their data, or biological material

Policy information about studies with human participants or human data. See also policy information about sex, gender (identity/presentation), and sexual orientation and race, ethnicity and racism.

Reporting on sex and gender	This study does not involve primary data collection from human participants, animals, or tissues, nor does it analyse sex- or gender-disaggregated outcomes. The analysis is based on a dynamic microsimulation model of Malawi's health system and population health. Where relevant, demographic and epidemiological parameters (e.g., disease incidence, mortality, health service utilisation) incorporate sex-specific patterns drawn from national data sources; however, the study does not focus on differential effects by sex or gender.
Reporting on race, ethnicity, or other socially relevant groupings	This study does not involve the collection or analysis of primary data disaggregated by race, ethnicity, or other socially defined population groupings. The modelling framework represents the population of Malawi using nationally representative demographic, epidemiological, and health system data, but does not explicitly stratify or report outcomes by race, ethnicity, socioeconomic status, or other equity-relevant characteristics. While the model accounts for variation in health outcomes by age and sex, it does not evaluate differential impacts across socially defined subpopulations.
Population characteristics	This study uses a dynamic, individual-based simulation model representing the population of Malawi. The simulated population is assigned covariate-relevant characteristics based on nationally representative demographic, epidemiological, and health system utilisation data. Key individual-level characteristics include age, sex, HIV status, tuberculosis status, malaria infection, and other disease conditions (including acute lower respiratory infections, diarrhoeal diseases, and non-communicable diseases). Health service interactions are modelled conditional on these characteristics and include histories of diagnosis, treatment initiation, adherence, and retention in care. For infectious diseases such as HIV, TB, and malaria, individuals are modelled with dynamic disease progression and treatment pathways consistent with national treatment guidelines. No genotypic or personally identifiable information was used or modelled. All inputs reflect population-level patterns rather than individual-level patient data.
Recruitment	NA
Ethics oversight	The Thanzi La Mawa project received ethical approval from the College of Medicine Malawi Research Ethics Committee (COMREC, P.09/23-0297) for the use of publicly accessible and anonymised secondary data. No data were used requiring individual informed consent.

Note that full information on the approval of the study protocol must also be provided in the manuscript.

Field-specific reporting

Please select the one below that is the best fit for your research. If you are not sure, read the appropriate sections before making your selection.

- [x] Life sciences
- [ ] Behavioural & social sciences
- [ ] Ecological, evolutionary & environmental sciences

For a reference copy of the document with all sections, see nature.com/documents/nr-reporting-summary-flat.pdf

Life sciences study design

All studies must disclose on these points even when the disclosure is negative.

Sample size	This study used the Thanzi La Onse simulation model, an integrated individual-based model of the health system and multiple health conditions. The number of datasets used was determined by the data requirements of the model structure, including demographic processes, epidemiological parameters, health-service utilisation, intervention coverage, health-system capacity, costs, and disease-specific calibration targets. Datasets were not selected to achieve a target sample size; rather, all datasets required to parameterise, calibrate, and validate the relevant components of the model were identified through the model development process and are listed in the accompanying model documentation and repository/website.<br>These datasets were considered sufficient because, collectively, they provide the empirical inputs needed to represent the population, health system, disease processes, intervention delivery, and calibration targets relevant to the analyses. Where multiple data sources were available for the same quantity, sources were prioritised according to relevance to the study setting, representativeness, recency, internal consistency, and suitability for model parameterisation. Remaining uncertainty in model inputs was represented through parameter uncertainty, calibration, and scenario analyses where appropriate.
Data exclusions	No individual-level participant data were collected for this study. The analysis used model inputs derived from anonymised aggregated datasets, publicly available datasets, published literature, and routinely collected programme or health-system data, as documented in the model repository/website.<br>Data exclusions, where applicable, occurred during model parameterisation and data harmonisation rather than through participant-level eligibility criteria. These included exclusion of duplicate records, internally inconsistent records, records outside the relevant geographical or temporal scope of the model, and data fields not required for the modelled quantities. Exclusion and cleaning decisions were made according to pre-specified data processing scripts or documented modelling assumptions. No data were excluded on the basis of observed model outcomes.
Replication	The analyses were conducted using open-source code from the Thanzi La Onse model. The version of the code corresponding to this manuscript is archived as a tagged GitHub release, and the model inputs used for the analyses, including model parameters, anonymised

	aggregated data and publicly available data, are available within the repository. Model documentation describes the parameter values, calibration methods, appointment footprints, and assumptions used in the analyses.<br>Reproducibility was assessed by rerunning the analysis pipeline from the archived code and input files, using the documented model configuration and random seeds where applicable. Outputs were checked against the manuscript results, including summary statistics, scenario comparisons, and generated figures/tables. All core findings were reproducible from the archived analysis code and data. Stochastic variation is expected in individual simulation runs, but replicated runs using the same code, inputs, configuration, and seeds reproduced the reported results within the expected numerical precision.
Randomization	This study did not involve allocation of participants or samples into experimental groups. The comparison groups were modelled scenarios, defined by assumptions about health-system conditions, intervention coverage, or programme implementation. These scenarios were applied within the same underlying model structure and population framework, allowing differences in outcomes to be attributed to the specified scenario assumptions rather than to differences in sampled populations.<br>Where stochastic simulations were used, scenarios were compared using the same model structure, input datasets, and, where appropriate, matched random seeds or repeated simulation draws to reduce stochastic variability. Covariates were controlled through the model parameterisation and calibration process, which represents demographic, epidemiological, and health-system characteristics explicitly within the simulation framework. Random allocation was therefore not relevant.
Blinding	Blinding was not relevant to this study. The analysis was based on a computational simulation model rather than an experiment involving human participants, animals, or laboratory samples. There was no group allocation during data collection. Scenario definitions were specified in advance as part of the modelling analysis, and model outputs were generated by deterministic code execution and/or stochastic simulation rather than by investigator assessment of participant outcomes. Therefore, investigator blinding to group allocation during data collection or analysis was not applicable.

Reporting for specific materials, systems and methods

We require information from authors about some types of materials, experimental systems and methods used in many studies. Here, indicate whether each material, system or method listed is relevant to your study. If you are not sure if a list item applies to your research, read the appropriate section before selecting a response.

Materials & experimental systems

n/a	Involved in the study	
☒	☐	Antibodies
☒	☐	Eukaryotic cell lines
☒	☐	Palaeontology and archaeology
☒	☐	Animals and other organisms
☒	☐	Clinical data
☒	☐	Dual use research of concern
☒	☐	Plants

Methods

n/a	Involved in the study	
☒	☐	ChIP-seq
☒	☐	Flow cytometry
☒	☐	MRI-based neuroimaging

Plants

Seed stocks	NA
Novel plant genotypes	NA
Authentication	NA
