# Supplementary material for: System-wide investments generate health and enhance HIV, TB and malaria control in Malawi
Source: Nat Commun. 2026 Sep 4;17:10541. doi: 10.1038/s41467-026-77306-5 (PMC13642251; doi:10.1038/s41467-026-77306-5)
Supplement: Supplementary file 3 [file 41467_2026_77306_MOESM3_ESM.pdf]

nature portfolio

Peer Review File

System-Wide Investments Generate Health And Enhance HIV, TB and Malaria Control in Malawi

Corresponding Author: Dr Tara Mangal

This file contains all reviewer reports in order by version, followed by all author rebuttals in order by version.

Version 0:

Reviewer comments:

Reviewer #1

(Remarks to the Author)
This is an interesting paper that attempts to get at one of the fundamental debates about the highly verticalized approach of global health in the last 20 years. I think it will be an important piece, even while I understand that models have the nature of oversimplifying the nature of phenomena and policy. However, there are reasons to be concerned that the oversimplification or lack of clarity about what is meant by vertical, horizontal, and diagonal interventions, as illustrated by this piece, may lead to more confusion.

The abstract greatly oversimplifies what is a diagonal approach, on p. 3, line 35. It is important to note that "diagonal investments" are not merely the combination of both horizontal and vertical. Please refer to Sepulveda et al 2006 for a careful reading of what diagonal approaches entail. This is the original usage of term "diagonal", a reference that has been curiously omitted from this paper (https://pubmed.ncbi.nlm.nih.gov/17141709/). Later on page 6 line 105, the authors indicate that diagonal approach involves expanding the interventions, which is indeed more similar to what Sepulveda called for. I skimmed through the reference by Kirwin et al. 2022, and it is not apparent to me that Sepulveda and Kirwin are aligned in their definition of diagonal. But I would not be surprised and indeed I would expect that the usage of the terms vertical (V), horizontal (H), and diagonal (D) differ depending on who is speaking. Thus, I think the authors need to be much more rigorous and explicit about what they think these terms V, H, and D are, relative to what the literature defines it as. They do define to some extent what they mean for their specific model inputs about what V, H, and D mean, but what is missing is a rigorous review of the literature on what this actually means.

Indeed, one of the most important impacts of a vertical approach is its negative impacts on other diseases, and not merely its fragmentation of the delivery. See this key paper by Grepin 2012 (https://www.healthaffairs.org/doi/full/10.1377/hlthaff.2012.0279) on how vertical approaches can negatively impact other diseases. If this is explicitly modeled in the paper, I do not see it. The mechanism by which these negative impacts can occur relate to the switching of health workers and service delivery from government systems to parallel, bilaterally financed delivery systems, often in the form of NGOs or even private sector, whose workers and patients may not even be on the books of the MOH. Thus, I question the use of the MOH roster of health workers as accurate. It is very possible that the MOH does not have the full roster of health workers who are working in these parallel delivery systems. The IOM PEPFAR evaluation from the 2010s indicated that ministries of health often have no idea what are the activities of bilateral donors in their own countries delivering these vertical services.

In short, I think the paper is a good attempt to try to unpack these thorny, nearly philosophical approaches, about what is V, H, and D, but their conclusions miss out on the complexity of what is V, H, and D, and what is the fundamental problem that each is trying to address and what are the unintended consequences or negative impacts of each. Even the term "V, H, and D" approaches vs investments is assumed to be the same; but I would argue that V, H, and D are approaches rather than investments. This may seem like a small terminological difference, but it's not just about the dollar amounts; it's about the ways the care is structured and delivered in public and private sectors with different financing sources.

Lastly, from the view of policy relevance, I would like to ask the authors what is their ultimate policy objective and decision desired by a policymaker. Do policymakers make decision solely on cost-effectiveness? If so, then they are fine to provide only the ICERs of these 3 policy options. But the reality is that there are at least a few other key factors that require consideration, which is about budgets, budget impact, whether we are talking about a theoretical budget increase or

decrease, or whether this is as an actual budget increase or decrease. I would strongly urge the authors to consider a practical scenario that countries are facing, which is to the effect of the following: Countries are facing cuts to the health sector, both from government budgets due to increasing debt burdens as well as cuts from aid, some of which shows up on government budgets and others which show up on NGO budgets and parallel service delivery systems. If we assume the majority of cuts are in the latter and that we are looking at the policymaker which is the government about what the government can do, then the question is, what tradeoffs do the policymaker have to make in order to have the fewest lives lost? Where will they find the efficiencies to cover the services that were previously covered by aid? Will health workers under the parallel system be re-absorbed at far lower salaries and be expected to deliver the same quality of care and access to care for the same or more set of services? I believe this would be a more practical and realistic question to what Malawi or another country would be facing, and how do they actually tackle this, and what role can modeling provide to make the best decisions for this. My greatest and regular annoyance with the modeling community and literature is their complete separation from actual policymaking decisions. While I know and see the value of modeling, I hope that modelers can better integrate and listen to what policymakers actually need. See this report and paper for example:
https://iris.who.int/handle/10665/376712
https://pubmed.ncbi.nlm.nih.gov/35627656/

Thank you for this opportunity to review this paper, and I wish the editors and authors good luck in revising it.

(Remarks on code availability)
n/a

Reviewer #2

(Remarks to the Author)
This paper is noteworthy in its evaluation of the health value and costs of vertical, horizontal and diagonal health system interventions. The motivation of the paper is well articulated and clear, and it identifies an important gap in the literature where the impact of horizontal and diagonal interventions are difficult to measure. The results are in line with my general understanding of the benefits of horizontal and diagonal investments versus vertical ones. The majority of my comments relate to the assumptions made in the models, how they affect results, and how a reader could interpret the extensibility of the results beyond the micro-simulation to Malawi or other settings. I organize my comments into major and minor comments below.

Major comments:
1. What is baseline? Is this if 2024 staff levels and resources were kept constant? Why wouldn't scale up of HRH staff consistent with 2017-2024 be considered baseline? This seems like a crucial decision, since the definition of whether an intervention is "vertical" or "diagonal" depends on the baseline HSS scenario. Is it a fair representation of vertical interventions to assume no investment in the health system over the period?
2. It was hard to understand where the assumptions between inputs and outcomes came from. For example increase in X% HRH leads to Y% reduction in DALYs, and whether the relationship was linear.
3. Timing question - in the discussion, you mention that HSS efforts are not as immediate as is assumed in the model. You could also hypothesize that immediate HSS efforts produce long-term benefits to population health which are realized slowly over time. It's unclear whether the TLO model incorporates this potential accumulating benefit, or whether it assumes steady-state outcomes dependent only on investments in the same period. The methodological point, and a discussion of its potential impact on the interpretation of results, would be useful.
4. The authors find diagonal intervention strategies to produce the greatest averted DALYs and the highest ROI. However, these hypothetical scenarios do not appear to be constrained to a particular budget, nor do they explicitly measure a tradeoff between investments in horizontal strategies over vertical or vice versa. Since this setting like other LMICs are resource-constrained, how meaningful are these results in the real world? Does this simply reflect that increasing investment along any axis is beneficial, and doubling investments is twice as good?

Minor comments:
5. It's unclear from the description of the TLO model how "appointment footprint" metrics are used to estimate health states. I would like to see more information on the model construction, especially in relation to the inputs and outputs, and what is held constant.
6. Please provide some basic information of the impact of the Covid-19 pandemic in Malawi, and whether it could have had impacts on the HRH or other data inputs.
7. Some concepts and terms - namely ASC, for example - are described in Results and Discussions but defined in Methods. Please provide enough information about these concepts earlier in the paper in the order that it's organized.
8. Definition of non-HTM diseases: i understood this to include acute LRI and diarrheal diseases, and no other disease. If this is so, then I suggest calling them as they are, otherwise it seems like it covers, literally, all non-HTMs, including all NCDs.
9. Are there empirical literature measuring the impact (health impact or C/E) of vertical programs? If so in the discussion section it would be useful to compare your results against empirical papers.
10. I am not sure whether the differences between the scenarios are considered substantial in real life. For example the ICER for the diagonal was $109 compared to the vertical $132. Is this difference large or small? I am asking because I do not know, and would be useful to have context around these numbers to interpret them. There is a strong political/economic reasons for GHIs implementing vertical programs, so it would be hard to promote diagonal unless there is a significant difference in outcomes (which I believe you show – 4x DALYs) or C/E.

(Remarks on code availability)

Reviewer #3

(Remarks to the Author)
I co-reviewed this manuscript with one of the reviewers who provided the listed reports. This is part of the Nature Communications initiative to facilitate training in peer review and to provide appropriate recognition for Early Career Researchers who co-review manuscripts.

(Remarks on code availability)

Reviewer #4

(Remarks to the Author)
This health system model-based economic evaluation is innovative, very well conducted and reported and has the potential to make a real difference in the way we think about priority setting for achieving UHC in LMICs. To achieve this in the current climate of aid cuts, however, it should change its pitch from addressing global health initiatives alone, to addressing both GHIs and LMIC governments. The rationale of the study (bottom of p.5) is based on the challenge of measuring the effects of health system strengthening investments, which the authors claim produces an evidence gap whose "effect of funding preferences threatens the efficiency, and effectiveness of global health investments". However, arguments in favour of horizontal investments are not new, and they come from a growing recognition of their importance built on the back of different kinds of evidence. Arguably, investment preferences towards vertical programmes are in no small part dictated by other factors, including the relatively short-term nature of donor funding (which in turn determines preference for impact metrics such as ROI), as well as donor preference for off-budget support. To make the premise of this work a bit less 'academic' and quickly outdated, given the rapid dismantling of the donor-led investment model predicated on GHIs, it would be good to acknowledge these issues with the outgoing model and the implications of the current climate of aid cuts in the introduction.

Given the above, there would be scope for reframing the findings as an 'investment case' for government-funded health systems strengthening/diagonal investments, to maximise the efficiency of whatever resources are available domestically. A minor related point is to define 'diagonal investments' in the abstract, as it is not (yet) a widely used term. Also relatedly, the authors may want to discuss among the limitations the fact that the base case used in the analysis considers no expansion of the health system's capacity in 2024, while it is quite possible that places like Malawi may go on to experience a resource contraction if government investment does not match donor withdrawal in the short to medium term.

Finally, I found the finding that horizontal investment on its own is always dominated by the vertical scenarios fascinating – but wondered about its wider applicability to other LMICs. This result would probably translate more directly if the authors could elaborate in a bit more practical detail on the 'vertical' HTM interventions that were modelled, as presumably some HTM interventions are more subject to both demand and supply constraints than others (e.g. new PrEP technologies vs scaling up of GeneXpert for TB). Ultimately, the balancing of horizontal vs vertical investment is likely also a factor of health system 'maturity', whereby more 'advanced' health systems may benefit from larger shares of horizontal investments compared to health systems with lower levels of government health financing that are still establishing and/or scaling up basic health services packages. Such practical insights and discussion of areas for future exploration probably deserve a mention in the discussion

(Remarks on code availability)

Version 1:

Reviewer comments:

Reviewer #2

(Remarks to the Author)
I have reviewed the updated manuscript, edits, and the response to the reviewers. The authors responded to the comments thoroughly. I have no further comments.

(Remarks on code availability)

Reviewer #3

(Remarks to the Author)
I co-reviewed this manuscript with one of the reviewers who provided the listed reports. This is part of the Nature Communications initiative to facilitate training in peer review and to provide appropriate recognition for Early Career Researchers who co-review manuscripts.

(Remarks on code availability)

Reviewer #5

(Remarks to the Author)
The authors have responded comprehensively and constructively to the earlier reviewer comments, particularly those raised by Reviewers 1 and 3. The revised manuscript is clearer, more precise, and better aligned with the decision problem it seeks to address. In particular, the move away from the potentially ambiguous language of vertical, horizontal and diagonal investments toward the more substantive categories of HTM-focussed programme scale-up, health system strengthening, and combined approaches is a sensible and effective revision. This change improves conceptual clarity without requiring the manuscript to become a definitional review of terminology that is not central to the paper's empirical contribution. The additions explaining finite system capacity, displacement effects, the scope of the MoH platform, and the relationship between service-specific and system-wide investments also strengthen the paper's interpretation and policy relevance.

The response to Reviewers 2-4 is similarly strong. The authors have taken seriously the need to speak not only to global health initiatives, but also to government decision-makers operating under constrained and potentially contracting fiscal conditions. The revised framing now better acknowledges donor funding mechanics, off-budget and parallel delivery channels, declining aid, and the importance of budget-informed investment sequencing - all realities in the current moment of global health. The addition of the cost-effectiveness frontier and expansion path provides a more practical basis for interpreting the findings under different resource envelopes, while the discussion of contraction risks and the companion Malawi analysis also helps situate the study within current policy realities. Overall, I find the responses sufficient, thoughtful, and well integrated into the revised manuscript.

(Remarks on code availability)

Open Access This Peer Review File is licensed under a Creative Commons Attribution 4.0 International License, which permits use, sharing, adaptation, distribution and reproduction in any medium or format, as long as you give appropriate credit to the original author(s) and the source, provide a link to the Creative Commons license, and indicate if changes were made.
In cases where reviewers are anonymous, credit should be given to 'Anonymous Referee' and the source.
The images or other third party material in this Peer Review File are included in the article's Creative Commons license,

unless indicated otherwise in a credit line to the material. If material is not included in the article's Creative Commons license and your intended use is not permitted by statutory regulation or exceeds the permitted use, you will need to obtain permission directly from the copyright holder.
To view a copy of this license, visit https://creativecommons.org/licenses/by/4.0/

REVIEWER COMMENTS

We thank the reviewers for their careful reading of the manuscript and for the constructive and insightful comments, which have helped to strengthen the clarity and rigour of the paper.

Reviewer #1 (Remarks to the Author):

This is an interesting paper that attempts to get at one of the fundamental debates about the highly verticalized approach of global health in the last 20 years. I think it will be an important piece, even while I understand that models have the nature of oversimplifying the nature of phenomena and policy. However, there are reasons to be concerned that the oversimplification or lack of clarity about what is meant by vertical, horizontal, and diagonal interventions, as illustrated by this piece, may lead to more confusion.

The abstract greatly oversimplifies what is a diagonal approach, on p. 3, line 35. It is important to note that "diagonal investments" are not merely the combination of both horizontal and vertical. Please refer to Sepulveda et al 2006 for a careful reading of what diagonal approaches entail. This is the original usage of term "diagonal", a reference that has been curiously omitted from this paper (https://pubmed.ncbi.nlm.nih.gov/17141709/). Later on page 6 line 105, the authors indicate that diagonal approach involves expanding the interventions, which is indeed more similar to what Sepulveda called for. I skimmed through the reference by Kirwin et al. 2022, and it is not apparent to me that Sepulveda and Kirwin are aligned in their definition of diagonal. But I would not be surprised and indeed I would expect that the usage of the terms vertical (V), horizontal (H), and diagonal (D) differ depending on who is speaking. Thus, I think the authors need to be much more rigorous and explicit about what they think these terms V, H, and D are, relative to what the literature defines it as. They do define to some extent what they mean for their specific model inputs about what V, H, and D mean, but what is missing is a rigorous review of the literature on what this actually means.

Response:
We thank the reviewer for raising this important conceptual point. We agree that the terms *vertical*, *horizontal*, and *diagonal* have been used inconsistently in the global health literature since the foundational framing by Sepúlveda et al. (2006), often leading to ambiguity about what each entails in practice.

In revising the manuscript, we carefully considered whether to retain this terminology. After discussion with our Ministry of Health partners and co-authors, we decided to remove the labels entirely and instead describe each scenario explicitly by its substantive content:

- *HTM-focussed programme scale-up* (analogous to vertical investment),
- *Health system strengthening (HSS)*, encompassing workforce and supply-chain improvements (analogous to horizontal investment), and
- *The combined approach*, representing integrated expansion of both programme-specific and cross-cutting capacities.

This decision was made precisely to avoid the definitional uncertainty the reviewer notes, while keeping our framing consistent with the intent of the “diagonal” concept as originally articulated by Sepúlveda et al. In this case, we decided not to conduct a rigorous review of the literature around these terms, as we are not looking to synthesise a broad set of definitions, but adhere to this clearer framing instead.

We have also included reference to this foundational paper in the Main section:

"*Early analyses also highlighted that investments targeted at priority diseases could be designed to strengthen underlying delivery functions rather than operate solely within programme silos (Sepúlveda et al).*"

We therefore now acknowledge this definitional issue in this response letter but have not reintroduced the terminology in the manuscript, since the revised formulation more clearly conveys the mechanisms modelled and prevents confusion.

Indeed, one of the most important impacts of a vertical approach is its negative impacts on other diseases, and not merely its fragmentation of the delivery. See this key paper by Grepin 2012 (https://www.healthaffairs.org/doi/full/10.1377/hlthaff.2012.0279) on how vertical approaches can negatively impact other diseases. If this is explicitly modeled in the paper, I do not see it.

Response: This mechanism is represented in our model, which allocates finite staff time and consumables across services. Expansion of one programme necessarily reduces resources available for others.

We have clarified this in the Methods by adding a new section (Constraints in health system supply) and added further details in the Discussion:

"Despite positive health gains for HIV-specific outcomes, overall DALYs and deaths did not significantly improve under the HIV Program scale-up without accompanying health system strengthening (0.44% [-0.02 – 1.37] increase in DALYs and 0.23% [-0.32 – 2.00] of deaths averted). In this model, these resources are finite and shared across programmes; increasing provision for HIV necessarily reduces the capacity available for other conditions, displacing or delaying care. This reflects the high baseline performance of the HIV care cascade and the modest short-term impact of expanding prevention activities, which, while resource-intensive, yield limited reductions in DALYs within the timeframe of this analysis. This finding, in which disease-focused investments can negatively affect non-target services, is consistent with empirical evidence that vertical programmes have sometimes diverted resources away from primary care."

The mechanism by which these negative impacts can occur relate to the switching of health workers and service delivery from government systems to parallel, bilaterally financed delivery systems, often in the form of NGOs or even private sector, whose workers and patients may not even be on the books of the MOH. Thus, I question the use of the MOH roster of health workers as accurate. It is very possible that the MOH does not have the full roster of health workers who are working in these parallel delivery systems. The IOM PEPFAR evaluation from the 2010s indicated that ministries of health often have no idea what are the activities of bilateral donors in their own countries delivering these vertical services.

Response:

We agree with the reviewer on the important issue of parallel, donor-financed delivery systems and their implications for workforce accounting. Our analysis is intentionally focussed on the public, Ministry of Health platform, because the policy question we address is how alternative investments strategies (HTM-focussed vs. health system strengthening) perform within the government system where budgetary and planning decisions are taken. Consistent with this scope, the Thanzi la Onse model represents funded MoH posts and allocates MoH staff time across services. This scope and data provenance are described in the Methods section and we have added the following text to address this point:

"The model represents service delivery through government and CHAM facilities, but excludes capacity from other private providers. Human resources are parameterised from MoH records of funded posts, and service capacity is generated by allocating patient-facing time from these cadres across services. Parallel, donor-financed NGO or private delivery platforms are not modelled as additional capacity unless operating through MoH facilities and captured in MoH routine data. "

As our objective is to inform MoH planning using MoH-managed resources, we consider the MoH roster the appropriate reference for the decision problem at hand. Nonetheless, we explicitly acknowledge that if large parallel services operate off-platform:
i) our estimates of displacement effects within MoH facilities could be upper-bounded (parallel capacity can partly buffer crowd-out in the public system); while,
ii) if NGO staff are seconded to or co-located within MoH facilities but not fully captured in MoH HRH data, the effective public capacity may be under-counted.

Given these countervailing mechanisms and the scarcity of harmonised, facility-level staffing data outside the MoH system, we caution against extrapolating across the full mixed health system. We now clarify the model's scope, justify the use of the MoH roster for our question, and state the direction of the potential bias as requested in the Discussion as follows:

"In Malawi, bilateral or NGO programmes provide services outside MoH administrative control, and these workers and patient volumes may be incompletely enumerated in MoH HRH and service statistics. For our MoH-centred investment strategy analysis, using the MoH roster is appropriate; however if substantial off-platform capacity exists, our estimates of crowd-out within MoH facilities may be upper bounds. Conversely, where NGO staff are seconded to MoH facilities but not fully recorded, effective public capacity may be under-counted. We therefore interpret displacement effects as public-platform effects, and caution against extrapolating to the entire mixed health system without additional data on parallel platforms. "

In short, I think the paper is a good attempt to try to unpack these thorny, nearly philosophical approaches, about what is V, H, and D, but their conclusions miss out on the complexity of what is V, H, and D, and what is the fundamental problem that each is trying to address and what are the unintended consequences or negative impacts of each. Even the term "V, H, and D" approaches vs investments is assumed to be the same; but I would argue that V, H, and D are approaches rather than investments. This may seem like a small terminological difference, but it's not just about the dollar amounts; it's about the ways the care is structured and delivered in public and private sectors with different financing sources.

Response:

We agree that what matters conceptually is the approach to organising and delivering care, not labels on "investments". We have therefore removed the references to vertical/horizontal/diagonal (as noted above) and consistently use HTM-focussed, HSS and combined. These denote approaches to structuring delivery which we operationalise in TLO as programme directives that change capacity (appointments and staff time), consumable availability and disease program coverage within the MoH platform.

'Vertical, horizontal and diagonal' are best understood as approaches to organising and governing care, not investments per se. Our analysis asks a narrower, decision-facing question: given the current capacity of the public health system, how do alternative investment strategies (aligned but not identical to V/H/D approaches) impact on population health? To make this evaluable, we used the TLO Model to map each approach to a bundle of investment that change service capacity, coverage and system readiness in MoH facilities. We have now made this distinction explicit, removing references to V/H/D that previously read as if V/H/D were synonymous with "investments'.

We have removed references to V/H/D strategies / investments. We have also added the following to the Main text:
"These investments describe how delivery is structured, whether resources and accountability are focussed in HTM programmes, in cross-cutting enablers, or in a combination, rather than denoting spend levels alone."

In the Methods we have added:
"We implement the three approaches as programme directives that determine where marginal MoH funds are placed and how service delivery is structured. HTM-focussed scenarios scale disease-specific interventions (HIV, TB, malaria) within prevailing system constraints. HSS scenarios expand cross-cutting enablers (HRH and consumable availability) that relax those constraints across services. Combined scenarios jointly expand HTM programmes and the HSS package. The model translates these directives into changes in human resource capacity (appointments, staff time), likelihood of consumables being available and coverage in MoH facilities and then simulates the health and economic outcomes."

Lastly, from the view of policy relevance, I would like to ask the authors what is their ultimate policy objective and decision desired by a policymaker. Do policymakers make decision solely on cost-effectiveness? If so, then they are fine to provide only the ICERs of these 3 policy options. But the reality is that there are at least a few other key factors that require consideration, which is about budgets, budget impact, whether we are talking about a theoretical budget increase or decrease, or whether this is as an actual budget increase or decrease.

Response:
Our primary objective is to contribute rigorous, quantitative evidence to the ongoing debate on the relative value for money of disease-focused versus broader system investments by explicitly estimating their comparative health impacts and cost-effectiveness within a constrained health system. This question remains central to global health financing and is a key consideration for both national planners and external funders. We fully agree that decision-making in practice is not based on cost-effectiveness alone, and that budget constraints, trade-

offs, and marginal investment choices are critical in determining real-world resource allocation.

To support this, we have now added an expansion path (added to Figure 2 and full expansion path shown in Supplementary Figure A1), constructed by identifying efficient scenarios along the cost-effectiveness frontier (as defined in Bertram et al., 2021). This additional, detailed analysis involved running a number of additional scenarios and allows policymakers to consider sequential investment options under different budget envelopes, providing additional practical guidance.

Further, our framework enables examination of both actual and theoretical budget changes. The expansion path can be interpreted either within a fixed budget (identifying the most efficient configuration of investments) or as evidence to support a case for increasing health sector resources by demonstrating the marginal health gains that additional funds could secure.

The text added to the manuscript now reads:

"*Further, to guide investment sequencing, we identified an expansion path by selecting the most efficient scenarios along the cost-effectiveness frontier, following WHO-CHOICE guidelines. The resulting path includes (1) Standalone TB program scale-up (2) TB program scale-up with consumables increased to the 75th percentile; (3) TB program scale-up with the full HSS Expansion Package; and (4) joint TB and malaria program scale-up with the full HSS Expansion Package. These scenarios represent progressively higher investment levels that deliver the greatest incremental health benefits per dollar spent (Supplementary Figure A.1).*"

This new result complements our main findings by grounding the cost-effectiveness comparisons in a more incremental, budget-informed policy framework, without distracting from the core message of the paper.

I would strongly urge the authors to consider a practical scenario that countries are facing, which is to the effect of the following: Countries are facing cuts to the health sector, both from government budgets due to increasing debt burdens as well as cuts from aid, some of which shows up on government budgets and others which show up on NGO budgets and parallel service delivery systems. If we assume the majority of cuts are in the latter and that we are looking at the policymaker which is the government about what the government can do, then the question is, what tradeoffs do the policymaker have to make in order to have the fewest lives lost? Where will they find the efficiencies to cover the services that were previously covered by aid? Will health workers under the parallel system be re-absorbed at far lower salaries and be expected to deliver the same quality of care and access to care for the same or more set of services? I believe this would be a more practical and realistic question to what Malawi or another country would be facing, and how do they actually tackle this, and what role can modeling provide to make the best decisions for this. My greatest and regular annoyance with the modeling community and literature is their complete separation from actual policymaking decisions. While I know and see the value of modeling, I hope that modelers can better integrate and listen to what policymakers actually need. See this report and paper for example: https://iris.who.int/handle/10665/376712
https://pubmed.ncbi.nlm.nih.gov/35627656/

Response:

Thank you for this important comment. We agree entirely that modelling should directly inform the real trade-offs faced by governments under fiscal contraction. This study was co-developed with the Malawi MoH to guide prioritisation with the sector, focussing on expansionary investment strategies under MoH control. As noted above, and recognised by all reviewers, this thorny question has indeed been central to many policy and funding decisions, and also now sits at the heart of debates about the future of direct support for disease-specific programs (such as Gavi and the Global Fund).

Policymakers increasingly confront the opposite scenario – shrinking fiscal space and withdrawal of off-budget aid, resulting in the need to now deliver more care within public facilities with reduced funding. A companion analysis (Molaro et al, *Plos medicine*, 2025, https://doi.org/10.1371/journal.pmed.1004488), undertaken within the same co-production framework, explicitly examines the health impact of declining external assistance in Malawi using the TLO model.

We now highlight this work to provide additional context for our related analysis in the introduction:
"Amid tightening fiscal space, modelling has been used to characterise the health consequences of declining aid and to identify protection and efficiency options (Molaro et al, 2025)."

In the Discussion, we also acknowledge contraction risks and direct readers to the companion Malawi-specific analysis:
"If domestic financing does not replace declining external support, near-term capacity could contract, implying our Baseline comparisons may overstate achievable coverage relative to a contracting trajectory (Molaro et al., 2025)."

More broadly, our modelling framework is designed to support decision-making under both expansion and austerity, allowing policymakers to explore which combinations of service integration, human-resource reallocation, and supply-chain efficiency yield the greatest health preservation when total resources fall. Through the Thanzi La Onse partnership, this co-production model has been embedded within Malawi's health planning processes to ensure direct policy relevance and capacity transfer.

Together, these analyses demonstrate how modelling can move beyond abstract optimisation to address precisely the kind of fiscal trade-offs and absorptive-capacity challenges described by the reviewer, thereby informing practical policy decisions in real-world budget settings.

Thank you for this opportunity to review this paper, and I wish the editors and authors good luck in revising it.

Reviewer #2 (Remarks to the Author):

This paper is noteworthy in its evaluation of the health value and costs of vertical, horizontal and diagonal health system interventions. The motivation of the paper is well articulated and clear, and it identifies an important gap in the literature where the impact of horizontal and diagonal interventions are difficult to measure. The results are in line with my general

understanding of the benefits of horizontal and diagonal investments versus vertical ones. The majority of my comments relate to the assumptions made in the models, how they affect results, and how a reader could interpret the extensibility of the results beyond the micro-simulation to Malawi or other settings. I organize my comments into major and minor comments below.

Major comments:
1. What is baseline? Is this if 2024 staff levels and resources were kept constant? Why wouldn't scale up of HRH staff consistent with 2017-2024 be considered baseline? This seems like a crucial decision, since the definition of whether an intervention is "vertical" or "diagonal" depends on the baseline HSS scenario. Is it a fair representation of vertical interventions to assume no investment in the health system over the period?

Response:
In our analysis, the Baseline is defined as holding MoH HRH patient-facing time, consumable availability and programme coverage at their observed 2024 levels throughout 2025-2035. This is stated in the introduction and figure legends.

We adopted this “status-quo” counterfactual for two reasons. First, it reflects the immediate planning starting point operationally, i.e. what happens without new policy actions to expand staffing or programme coverage. Second, using a fixed 2024 baseline avoids incorporating HSS growth or HTM expansion into the counterfactual, which would obscure the incremental effects and value-for-money of explicit policy options. Trajectories that continue the historical 2017-2024 scale-up into future years are therefore treated as HSS scenarios, one option of a number of possible future expansion choices. This allows us to assess the marginal impact of investing in HSS versus disease programs, and the additional benefits achieved when the two are combined.

This approach does not imply no maintenance investment. Given our decision question, how a policy-maker should allocate new MoH-managed resources across HTM-focussed, HSS or combined options, we consider this Baseline appropriate and transparent.

We have added the following to the Methods:
“A Baseline scenario holds MoH service capacity and HTM coverage targets fixed at their 2024 levels for 2025-2035.”

2. It was hard to understand where the assumptions between inputs and outcomes came from. For example increase in X% HRH leads to Y% reduction in DALYs, and whether the relationship was linear.

Response:
Our results are derived from the Thanzi La Onse (TLO) model, a mechanistic, individual-based simulation that explicitly represents healthcare system constraints, such as workforce availability and supply chain performance, and their impact on service delivery and health outcomes, alongside the processes of disease progression, and transmission for diseases and conditions that account for the vast majority of ill health in this setting.

Thus, the relationships between inputs (e.g. HRH scale-up) and outcomes (e.g. DALYs averted) are not based on assumed proportional or linear relationships, but rather emerge from the

model's dynamic mechanistic structure. This incorporates assumptions about the patient-facing time required to deliver different services, the treatment effects of successfully delivered interventions, healthcare-seeking behaviours, and other real-world constraints, such as drugs being stocked out, that can prevent service delivery. The model also captures knock-on effects from delayed or denied treatments, which influence downstream morbidity and mortality. We explain this in more detail in the Method section (under sub-sections - *Study design and model overview, Health system structure, HIV, tuberculosis and malaria (HTM) programs*) and refer readers to Hallett et al. (2025) which provides a more detailed description of the mechanics and assumptions of the model.

3. Timing question - in the discussion, you mention that HSS efforts are not as immediate as is assumed in the model. You could also hypothesize that immediate HSS efforts produce long-term benefits to population health which are realized slowly over time. It's unclear whether the TLO model incorporates this potential accumulating benefit, or whether it assumes steady-state outcomes dependent only on investments in the same period. The methodological point, and a discussion of its potential impact on the interpretation of results, would be useful.

Response:

We agree with the reviewer on this important point regarding the temporal dynamics of system strengthening effects.

In TLO, benefits do accumulate over time, but not all channels are modelled. Specifically, (i) the model is dynamic and captures time-propagating effects via transmission, disease progression, ageing and repeated care use, so gains from HSS (e.g. higher coverage) accrue beyond the investment year; (ii) HRH scale-up is phased (2017-2024 fitted, 2025-2030 growth applied, then levels held constant), so capacity persists and continues to generate health gains beyond the ramp-up; and (iii) consumable availability changes persist once applied. This is stated in the Methods. However, we do not model endogenous improvements in productivity / quality from training or organisational learning; worker productivity is assumed fixed, and we implement an immediate utilisation response once capacity / availability is raised. Consequently, the model may overstate short-term gains by assuming an immediate response to increased capacity, and understate longer-term improvements that would arise from gradual enhancements in quality and productivity.

We now state this more clearly and discuss how it affects interpretation.

We have redrafted a paragraph in the Discussion to highlight these points:
"While our model accounts for several important constraints within the health system, there remain additional factors such as weak information systems, inadequate infrastructure, and governance challenges that could influence both the effectiveness and wider applicability of HSS efforts. Although we model the expansion of the health workforce and improvements in supply chains as mechanisms to increase service coverage, it is important to recognise that the success of these measures also depends heavily on factors such as the quality of training, staff retention, and the equitable distribution of personnel across different regions and levels of care… In this framework, some benefits accumulate over time via transmission, disease progression and repeated care use, but we do not represent gradual improvements in productivity or quality that might arise from enhanced training or supervision. As a result, the timing of gains may appear earlier than would appear in practice, whereas longer-term

improvements that accrue slowly through sustained quality enhancement may be understated."

4. The authors find diagonal intervention strategies to produce the greatest averted DALYs and the highest ROI. However, these hypothetical scenarios do not appear to be constrained to a particular budget, nor do they explicitly measure a tradeoff between investments in horizontal strategies over vertical or vice versa. Since this setting like other LMICs are resource-constrained, how meaningful are these results in the real world? Does this simply reflect that increasing investment along any axis is beneficial, and doubling investments is twice as good?

Response:
This comment raises an important issue about the interpretation of the diagonal scenarios in a resource-constrained setting. Our intention was not to suggest that "more investment is always better," but rather to compare the value for money of competing investment types – disease-focussed vs. health system strengthening vs. combined - under the assumption of additional available resources, as would be the case when designing strategic investment plans or prioritising donor contributions. Our scenarios are costed and compared on incremental cost-effectiveness and return on investment, they go beyond a simple mechanism by which benefits increase proportionally with higher levels of investment.

To really explore the benefits of expansion in key parts of the health system, we have now repeated the full analysis incorporating a number of additional simulations which we use to produce a full cost-effectiveness frontier so that an expansion pathway (over a wide range of budgets) can be reported.

Our updated Results section states the following (Section - Results: Value for money) –
"Among the three alternative joint investment scenarios, HTM program scale-up is therefore the least efficient use of resources. It is dominated by the HSS Expansion Package – being more costly and less effective - and it is excluded by extended dominance when compared to the combined strategy - HTM program scale-up with HSS Expansion. This is demonstrated by the expansion path (Figure 2), which selects the non-dominated scenarios that provide the greatest incremental health gains per dollar spent, following WHO-CHOICE guidelines."

This directly addresses the reviewer's point about constrained choice. The policy guideline here is - when fiscal space is available, first prioritise HSS Expansion; and then invest in HTM programme scale-up for the most efficient resource use. The pattern reflects non-linear interactions and binding constraints, not a mechanical payoff to "more spending".

Further we have now also explicitly identified an expansion path (Supplementary Figure A1), constructed from the most efficient constituents of these three joint strategies.
We have added the following to the results section:

"Further, recognising the need to account for limited fiscal space when sequencing investments, we decompose the broader investment strategies into five independent constituent options - HIV programme scale-up, TB programme scale-up, malaria programme scale-up, consumables increased to the 75th percentile, and historical HRH scale-up - and evaluate all 31 possible combinations of these against the baseline. The resulting expansion path comprises: (1) Standalone TB program scale-up (2) TB program scale-up with consumables increased to the 75th percentile; (3) TB program scale-up with the full HSS

Expansion Package; and (4) joint TB and malaria program scale-up with the full HSS Expansion Package. These scenarios represent progressively higher investment levels that deliver the greatest incremental health benefits per dollar spent (Supplementary Figure A.1)."

Minor comments:

5. It's unclear from the description of the TLO model how "appointment footprint" metrics are used to estimate health states. I would like to see more information on the model construction, especially in relation to the inputs and outputs, and what is held constant.

Response:

In TLO, an appointment footprint specifies the resources required for one completed contact between a patient and the healthcare system(minutes by cadre, diagnostics and consumables). These footprints are applied at facility level against patient-facing minutes and consumable availability.

We have added the following text in the methods to make this clear:

"An appointment footprint defines the resources for a single completed contact: (i) cadre-specific minutes, (ii) any diagnostics required, and (iii) consumables / medicines."

For completeness, we also note that the full model structure, including all appointment footprints and care pathways, is publicly available and documented in the open-source TLO model repository, should readers wish to examine the construction and assumptions in greater detail. We have included references to these in the Methods section.

6. Please provide some basic information of the impact of the Covid-19 pandemic in Malawi, and whether it could have had impacts on the HRH or other data inputs.

Response:

We did not explicitly model COVID-19. Our inputs (MoH funded posts, routine logistics data, programme targets) are taken from the most recent post-pandemic period and represent the current steady state rather than the 2020-2022 disruption. Any lasting consequences of the pandemic that persist in routine systems (e.g. changes in funded posts or revised stock ordering practices) are thus implicitly embedded in the 2024 baseline inputs, but we do not simulate COVID waves or acute service interruptions. We now state this explicitly and add a brief interpretive caveat.

Methods:

"COVID-19-related disruptions are not modelled here. Baseline HRH and supply inputs reflect a non-pandemic steady state, representing current conditions rather than acute 2020-2022 disruption."

7. Some concepts and terms - namely ASC, for example - are described in Results and Discussions but defined in Methods. Please provide enough information about these concepts earlier in the paper in the order that it's organized.

Response:

In line with the article structure required by the journal, we had to preserve the order of the Results and Methods section. We have removed this acronym (ASC) as it is not commonly used. We have now also added the following brief statement in the Value for Money sub-section under Results to define this term:
“Note that these estimates reflect only service-level costs, consistent with practical recommendations for cost-effectiveness analysis (Claxton et al, 2014), and exclude above service-level costs such as programme management and outreach.”

8. Definition of non-HTM diseases: i understood this to include acute LRI and diarrheal diseases, and no other disease. If this is so, then I suggest calling them as they are, otherwise it seems like it covers, literally, all non-HTMs, including all NCDs.

Response:

We agree with the reviewer that we could make this clearer. We now state explicitly that “non-HTM” covers all causes other than HIV, TB and malaria (including NCDs, maternal, neonatal and child health and injuries etc.). To avoid implying a narrow scope we have relabelled this as “All other causes (non-HTM)”.

9. Are there empirical literature measuring the impact (health impact or C/E) of vertical programs? If so in the discussion section it would be useful to compare your results against empirical papers.

Response:

We appreciate the reviewer’s request for comparison with existing empirical literature. Among published analyses, the WHO-CHOICE study by Ralaidovy et al. (2021) is the most directly comparable in terms of methodological intent, as it applies a generalised cost-effectiveness analysis (GCEA) framework across HIV, TB, and malaria interventions. However, meaningful direct comparison remains limited due to important differences in assumptions, methods, and context.

First, our study uses the Thanzi La Onse (TLO) microsimulation model, which is highly tailored to the Malawian health system and explicitly incorporates constraints such as workforce shortages, supply chain gaps, and service coverage dynamics. We estimate health and cost outcomes over an 11-year horizon (2025–2035), using local cost data and a health system (provider) perspective. In contrast, Ralaidovy et al. simulate each disease area using separate models over a 100-year horizon, adopt an all-payer perspective and do not systematically account for national system constraints.

After adjusting for inflation and purchasing power differences, the ICERs reported in Ralaidovy et al. translate to approximately USD 40–60 per DALY averted in 2023 USD terms for Malawi. Our estimates for integrated programmatic scale-up (in which health system capacity expands alongside programmatic scale up) range from USD 24.85 to USD 69.43. These figures are broadly comparable in magnitude, but, given the different modelling structures, time horizons, baseline assumptions, and cost perspectives, we did not think it was appropriate to make this comparison in the manuscript.

10. I am not sure whether the differences between the scenarios are considered substantial in real life. For example the ICER for the diagonal was $109 compared to the vertical $132. Is this difference large or small? I am asking because I do not know, and would be useful to have context around these numbers to interpret them. There is a strong political/economic reasons for GHIs implementing vertical programs, so it would be hard to promote diagonal unless there is a significant difference in outcomes (which I believe you show – 4x DALYs) or C/E.

Response:
As the scenarios we present involve non-marginal investments, we do not assess ICERs against a static cost-effectiveness threshold. Instead, we compare the relative efficiency of alternative investment options. As stated in the results section, the diagonal (now called "combined scenario") has a **49% lower** ICER than the vertical scenario (now called the "HTM-focussed scenario"). This can be interpreted as a near doubling of efficiency (i.e. generating health at half the cost).

For context, recent estimates place Malawi's cost-effectiveness threshold (CET) between $140–160 per DALY averted (Lomas et al., 2024), while the current Health Sector Strategic Plan III (HSSP-III) uses a more conservative figure of $65.80. However, because the final incremental investment in our diagonal scenario would involve a significant (**non-marginal**) budget expansion, comparing these ICERs with a static CET representing the marginal productivity (cost-effectiveness) of the current system would not be appropriate.
Further, by now including an expansion path to guide the most efficient sequencing of options, we provide clear policy guidance.
The following statements in the Results section support this interpretation –

1. *"Combined approaches consistently outperformed all HTM-focussed approaches, reducing DALYs by 15.03% (UI: 14.46 – 15.94%) compared to a 4.98% reduction through vertical investments alone"*
2. *"Combining the two approaches resulted in even greater health gains (16.17 (UI: 15.63 – 17.23) million DALYs averted), albeit at a greater cost ($1.08 (UI: $1.06 - $1.08) billion), yielding an ICER of $66.53 (UI: $62.60 - $67.91) per DALY averted. This represents a 49% reduction in cost per DALY averted relative to the HTM-focussed approach."*
3. *"Further, recognising the need to account for limited fiscal space when sequencing investments, we decompose the broader investment strategies into five independent constituent options - HIV programme scale-up, TB programme scale-up, malaria programme scale-up, consumables increased to the 75th percentile, and historical HRH scale-up - and evaluate all 31 possible combinations of these against the baseline. The resulting expansion path comprises: (1) Standalone TB program scale-up (2) TB program scale-up with consumables increased to the 75th percentile; (3) TB program scale-up with the full HSS Expansion Package; and (4) joint TB and malaria program scale-up with the full HSS Expansion Package. These scenarios represent progressively higher investment levels that deliver the greatest incremental health benefits per dollar spent (Supplementary Figure A.1)."*

Reviewer #3 (Remarks to the Author):

I co-reviewed this manuscript with one of the reviewers who provided the listed reports. This is part of the Nature Communications initiative to facilitate training in peer review and to provide

appropriate recognition for Early Career Researchers who co-review manuscripts.

Response:
We thank Reviewer #3 for their contribution to the review process.

Reviewer #4 (Remarks to the Author):

This health system model-based economic evaluation is innovative, very well conducted and reported and has the potential to make a real difference in the way we think about priority setting for achieving UHC in LMICs. To achieve this in the current climate of aid cuts, however, it should change its pitch from addressing global health initiatives alone, to addressing both GHIs and LMIC governments.

Response:
Thank you for this helpful suggestion. We have responded to a similar point made by Reviewer 1 above. We agree that policy relevance requires speaking to both LMIC governments and GHIs/donors. We believe that the revised manuscript does so: the newly added cost-effectiveness frontier and the expansion path are framed explicitly for a government budget-holder, indicating how to sequence HTM-focussed, HSS and combined options within fiscal limits. The same results also inform donors about efficient alignment / co-financing once minimum system capacity is in place.

The rationale of the study (bottom of p.5) is based on the challenge of measuring the effects of health system strengthening investments, which the authors claim produces an evidence gap whose "effect of funding preferences threatens the efficiency, and effectiveness of global health investments". However, arguments in favour of horizontal investments are not new, and they come from a growing recognition of their importance built on the back of different kinds of evidence. Arguably, investment preferences towards vertical programmes are in no small part dictated by other factors, including the relatively short-term nature of donor funding (which in turn determines preference for impact metrics such as ROI), as well as donor preference for off-budget support. To make the premise of this work a bit less 'academic' and quickly outdated, given the rapid dismantling of the donor-led investment model predicated on GHIs, it would be good to acknowledge these issues with the outgoing model and the implications of the current climate of aid cuts in the introduction.

Response:
We agree with the reviewer's points. We have revised the introduction to acknowledge the ongoing donor-led model and the current climate of aid contraction. We have also clarified that our contribution is to provide , government-relevant comparative evidence on HTM-focussed, HSS and combined options within the public platform given finite fiscal space.

We have added to the Main section:
"Historical preferences for HTM-focussed programmes have also reflected funding mechanics – notably short donor cycles with an emphasis on near-term, measurable impacts and the use of off-budget or parallel delivery channels – which shape what is financed and how it is implemented."

And

“Arguments for system-strengthening are longstanding and supported by multiple evidence streams.”

In response also to Reviewer 1, we have added a mention of the current funding conditions:
“*Amid tightening fiscal space, modelling has been used to characterise the health consequences of declining aid and to identify protection and efficiency options.”*

In the Discussion we also acknowledge contraction risks and direct readers to the companion Malawi-specific analysis:
“If domestic financing does not replace declining external support, near-term capacity could contract, implying our Baseline comparisons may overstate achievable coverage relative to a contracting trajectory (Molaro et al., 2025).”

Given the above, there would be scope for reframing the findings as an ‘investment case’ for government-funded health systems strengthening/diagonal investments, to maximise the efficiency of whatever resources are available domestically.

Response:
We agree that the results lend themselves to an investment-case framing for domestic budgeting. Our primary aim here is a novel and fair evaluation. We do expect, however, that this important addition to the evidence base will be important in many decisions for future investment at national and international levels.

A minor related point is to define ‘diagonal investments’ in the abstract, as it is not (yet) a widely used term.

Response:
For the sake of clarity, we have now avoided the term “diagonal” and use “combined approach” throughout.

Also relatedly, the authors may want to discuss among the limitations the fact that the base case used in the analysis considers no expansion of the health system’s capacity in 2024, while it is quite possible that places like Malawi may go on to experience a resource contraction if government investment does not match donor withdrawal in the short to medium term.

Response:
As noted in our earlier response to Reviewer 2, the Baseline holds 2024 service capacity and HTM programme coverage constant over 2025-2035. We agree this represents a Status Quo counterfactual rather than a contraction trajectory which may be one possible outcome of funding cuts. We have referenced in the Main text, a complementary analysis which does examine the implications of funding cuts in some detail (Molaro et al, 2025).

Main:
"Amid tightening fiscal space, modelling has been used to characterise the health consequences of declining aid and to identify protection and efficiency options."

We have also added the following in the Discussion:
"If domestic financing does not replace declining external support, near-term capacity could contract, implying our Baseline comparisons may overstate achievable coverage relative to a contracting trajectory."

Finally, I found the finding that horizontal investment on its own is always dominated by the vertical scenarios fascinating – but wondered about its wider applicability to other LMICs. This result would probably translate more directly if the authors could elaborate in a bit more practical detail on the 'vertical' HTM interventions that were modelled, as presumably some HTM interventions are more subject to both demand and supply constraints than others (e.g. new PrEP technologies vs scaling up of GeneXpert for TB). Ultimately, the balancing of horizontal vs vertical investment is likely also a factor of health system 'maturity', whereby more 'advanced' health systems may benefit from larger shares of horizontal investments compared to health systems with lower levels of government health financing that are still establishing and/or scaling up basic health services packages. Such practical insights and discussion of areas for future exploration probably deserve a mention in the discussion

Response:
We fully agree with the reviewer on this point. The full set of HTM-interventions is presented in Extended Data Table 1. To make the concept of "supply-side readiness" more tangible, we have added a new heatmap of consumables availability for specific HIV, TB and malaria-related consumables across facility levels 1a, 1b, 2 and 3. This directly demonstrates the heterogeneities in supply constraints across programmes.

We now reference this in the Results section (Health systems strengthening investments):

"Baseline consumables availability varies substantially across programmes and facility levels (Extended Data Figure1); consequently HSS improvements translate into non-linear improvements across multiple programmes."

Our analysis focuses on Malawi, but we recognise that the mechanisms we identify will play out differently in settings where core service platforms and delivery platforms are at more or less advanced stages of development, influencing how additional resources translate into service gains. We have added the following to the Discussion to highlight how the implications of disease-focussed versus system strengthening depend on the type of constraint and the maturity of the health system:

"Different HTM components are limited by different constraints, making the marginal return to HSS inherently intervention-specific. For supply-constrained services (e.g. TB diagnosis reliant on GeneXpert or chest x-ray), additional staff capacity and more reliable stocks yield larger gains. For demand-sensitive services (e.g. PrEP for HIV), demand-generation is the binding constraint, with smaller pay-offs from supply-side HSS alone. Although the analysis focuses on Malawi, the mechanisms identified are relevant across other LMICs. The balance between disease-focussed and HSS investments is expected to differ with the maturity and capacity of

individual health systems. In less-developed systems, disease-focussed scale-up can remain highly effective until foundational service delivery capacities are in place; as systems strengthen, the relative gains from further expansion diminish and broader HSS investments become increasingly valuable. Future comparative analyses could help quantify this transition and clarify how system development shapes optimal investment strategies."
